# Confinement of Polariton Condensates in Quasi‐Flat Band BICs in Plasmonic and Dielectric Metasurfaces

**DOI:** 10.1002/nap2.70092

**Published:** 2026-04-20

**Authors:** Anton Matthijs Berghuis, Jose Luis Pura, Rafael P. Argante, Shunsuke Murai, José A. Sánchez‐Gil, Jaime Gómez Rivas

**Affiliations:** ^1^ Department of Applied Physics and Science Education and Eindhoven Hendrik Casimir Institute Eindhoven University of Technology Eindhoven the Netherlands; ^2^ Institute for Complex Molecular Systems‐ICMS, Eindhoven University of Technology Eindhoven the Netherlands; ^3^ Instituto de Estructura de la Materia (IEM‐CSIC), Consejo Superior de Investigaciones Científicas Madrid Spain; ^4^ GdS‐Optronlab Física de la Materia Condensada, Universidad de Valladolid Valladolid Spain; ^5^ Department of Physics and Electronics, Graduate School of Engineering Osaka Metropolitan University Osaka Japan

## Abstract

We investigate exciton–polariton condensation in square arrays, composed of either dielectric silicon (Si) or plasmonic silver (Ag) nanodisks, covered with a dye‐doped layer. Both arrays support symmetry‐protected bound states in the continuum (BICs) at normal incidence, featuring electric quadrupolar $\left(Q_{xy}\right)$ and magnetic dipolar $\left(m_{z}\right)$ characters. Due to differences in mode coupling, these BICs are split by ∼10 meV in the Si array, whereas they remain nearly degenerate in the Ag array. Simulations reveal that interference in the Ag array results in hybrid modes, $\left(m_{z}+i{\tilde{Q}}_{xy}\right)$ and $\left(m_{z}-i{\tilde{Q}}_{xy}\right)$, which are polarized along orthogonal directions. Interestingly, this results in similar condensation thresholds in both Si and Ag arrays, regardless of the inherent nonradiative losses of Ag, and the confinement of the polariton condensates in the Ag array. Although condensation in the Si array occurs in the $Q_{xy}$ BIC, producing a characteristic donut‐shaped far‐field emission in k‐space, condensation in the Ag array populates the hybrid modes, leading to a double‐cross emission pattern extending over a broad range of wave vectors due to the quasi‐flat band nature of this mode. As a result, the Ag array also exhibits a strong confinement along the polarization axis in real space. However, for unpolarized emission, there is a similar spatial confinement in both Si and Ag arrays. This control over the confinement of condensates could also be exploited to control interactions. Our results highlight a novel mechanism for condensate confinement with potential applications in quantum computing and polaritonic circuitry.

## 1 Introduction

Exciton–polaritons are hybrid light–matter quasiparticles that can undergo stimulated relaxation into a single quantum state, resulting in coherent laser‐like emission. This phenomenon is known as polariton lasing or nonequilibrium polariton condensation [1, 2, 3, 4]. Due to their matter component, polaritons can interact with each other [1, 5, 6], as well as with other condensates [7], enabling a range of nonlinear and collective phenomena [8, 9]. This property not only makes polariton condensates an interesting platform to explore nonequilibrium many‐body physics but also promising candidates for quantum simulation and computation [10, 11, 12]. A key requirement for such applications is the ability to control and confine the condensate in both real and reciprocal space.

In recent years, there have been significant advances in the research of lasing from BICs and quasi‐BICs [13, 14, 15, 16, 17, 18, 19, 20, 21, 22], as well as polariton condensation [9, 23, 24, 25, 26]. BICs offer long radiative lifetimes as a result of their symmetry‐protected decoupling from free‐space modes, making them ideal cavities for condensation. Furthermore, BICs have topological charges [27, 28] and support complex polarization textures in both the near‐ and far‐field [29, 30]. However, the dispersion of the modes that define quasi‐BICs, which lead to BICs at the Γ−point (normal to the surface), and their concomitant high group velocities in extended metasurfaces often hinder the confinement of polaritons and increase the condensation threshold [30]. Various approaches have been developed to trap polariton condensates, including patterned microcavity arrays [31, 32] such as Lieb lattices [33, 34, 35]. In addition, optically defined traps [36, 37] and finite‐sized cavities [38] have been used. Another emerging method is the use of flat or quasi‐flat bands in metasurfaces, which can reduce group velocities and localize modes without requiring additional patterning [39, 40, 41]. In addition, engineering the cavity geometry allows for control over the polarization state and the multipolar character of the condensate, enabling further tuning of its interaction properties.

In this study, we demonstrate polariton condensation into a bound state in the continuum (BIC) in a quasi‐flat band supported by a plasmonic silver (Ag) metasurface. By combining real‐space and momentum‐space imaging, we show that the condensate exhibits strong confinement in real space along one axis for a single polarization state of the condensate. Although a dielectric silicon (Si) metasurface with similar geometry also supports a BIC, this mode is more dispersive, resulting in a condensate that is delocalized in real space but exhibits a narrow distribution in momentum space. We further provide a physical understanding of the distinct dispersions observed in the Ag and Si arrays. By performing a multipolar decomposition of the BICs, we show that the difference arises from the character of the modes in which the condensates are formed. In the Si array, condensation takes place in a quadrupolar mode, whereas in the Ag array, condensation occurs in two orthogonal modes $m_{z}\pm i{\tilde{Q}}_{xy}$. This different hybridization determines the polariton dispersion and polarization properties. Despite the intrinsic material losses of plasmonic systems, very similar condensation thresholds are achieved with the Ag and Si metasurfaces. These results highlight the relevance and strong impact that the polariton dispersion has on the condensation and provide new insights into the design of metasurfaces for polariton confinement, setting the stage for future studies of polariton–polariton interactions in metasurfaces.

## 2 Results

We have designed two square arrays, both with a period of 420 nm, consisting of disks of either Si or Ag nanodisks on top of a quartz substrate using electron beam lithography (see Supporting Information S1: Section S1). A single unit cell is schematically depicted in Figure 1a. To compensate for the intrinsicly larger scattering cross section of metal (plasmonic) nanoparticles compared to that of dielectric (Mie) particles, the Ag nanodisks (diameter = 50 nm, height = 60 nm) have smaller dimensions than their Si counterparts (diameter = 90 nm, height = 90 nm), as is also visible in the SEM images of the Si and Ag arrays in Figure 1c,d, respectively. These dimensions were chosen such that the quality factor (Q) of the bright surface lattice resonance of both structures yields approximately the same value (Si: Q∼200, Ag: Q∼150, based on simulations (see Supporting Information S1: Section S2). Additionally, the Ag nanoparticle arrays have a 2 nm $TiO_{2}$ adhesion layer and a 20 nm $Al_{2}O_{3}$ protective layer to prevent oxidation (see Supporting Information S1 for fabrication details).

**FIGURE 1:**
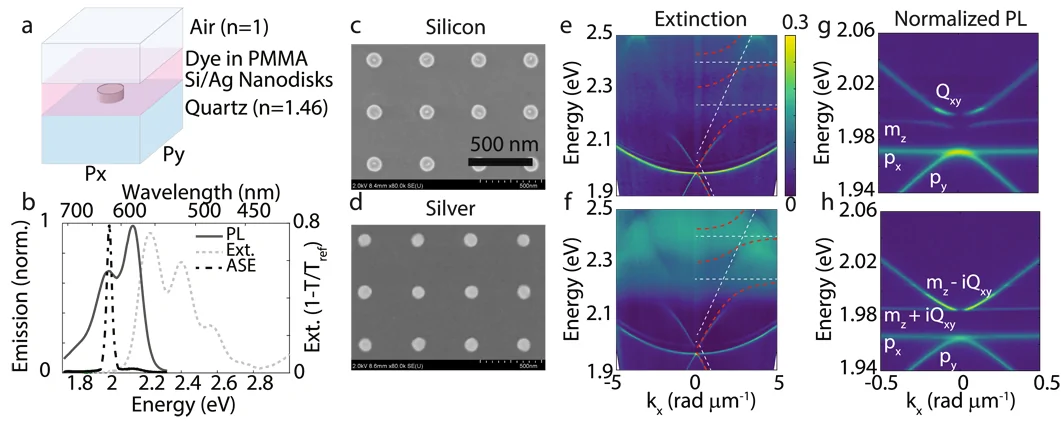
(a) Schematic geometry of the samples. Nanodisks from either Ag or Si are fabricated on a quartz substrate using e‐beam lithography in a square array. The nanodisks are covered with a layer of PMMA with a thickness of 220 nm doped with perylene dye molecules at a concentration of 30 wt%. (b) Extinction (light‐gray dashed curve), emission (solid‐black curve), and amplified spontaneous emission (dark‐gray dash–dotted curve) of the bare molecules. (c) and (d) SEM image of the Si and Ag nanoparticle arrays, respectively. (e) and (f) dispersion obtained by extinction measurements of the Si and Ag metasurfaces, respectively, where the white dashed lines indicate the SLRs and the exciton transitions. Both systems are in the strong coupling regime, as evidenced by the anticrossing of the metasurface mode with the lowest energy exciton. The red dashed curves are the calculated polariton dispersions from the coupling of the SLRs and two excitons. (g) and (h) Close‐ups of the photoluminescence (PL) from the strongly coupled metasurfaces near $k_{x}$ = 0. Both, the Si and Ag metasurfaces show a BIC at normal incidence.

As in our previous work [42], the nanoparticle arrays are covered with a ∼ 220 nm thick layer of dye‐doped PMMA (30% of perylene dye [N, N′‐Bis(2,6‐diisopropylphenyl)‐1,7‐ and −1,6‐bis (2,6‐diisopropylphenoxy)‐perylene‐3,4:9,10‐tetracarboximide] in PMMA). We use an organic dye because the low dielectric screening results in tightly bound Frenkel excitons that are stable at room temperature. This perylene dye has a large quantum yield and high solubility in PMMA without forming aggregates [4]. The absorption and emission spectra are shown in Figure 1b, where the light‐gray dashed curve is the absorption spectrum, the solid‐black curve is the emission spectrum, and the dark‐gray dash‐dotted curve is the amplified spontaneous emission (ASE), under intense pulse laser excitation (data reproduced from ref. [42]).

The extinction of the samples, defined as $1-T/T_{\mathrm{ref}}$, where T is the transmission through the sample and $T_{\mathrm{ref}}$ is the transmission of the layer stack without the nanoparticles, is characterized in a Fourier microscope (see Supporting Information S1: Section S3 for setup details), allowing to measure the band structure up to 37° with respect to normal incidence. The Fourier plane is imaged onto a slit and dispersed in energy, resulting in the band structures along the in‐plane wave vector $k_{x}$, as shown in Figure 1e for the Si array and Figure 1f for the Ag array. At normal incidence, the surface lattice resonances (SLRs) at ∼ 2 eV are red‐detuned from the Perylene main exciton resonance at 2.24 eV (both indicated with the white dashed lines in Figure 1e,f). The coupling between the excitons and SLRs can be described by a coupled oscillator model, resulting in the polariton dispersion as indicated with the red‐dashed curves in Figure 1e,f. At the energy of the BIC, the exciton fraction of the polariton, obtained from the Hopfield coefficients, is approximately 10% for both the Si and Ag arrays. The details of the coupled oscillator model are given in the Supporting Information S1: Section S13. It should be noted that the unpolarized dispersions of the Si and Ag samples appear similar in Figure 1e,f, as the particle sizes were deliberately tuned to achieve comparable scattering cross sections. When we, however, take a closer look at the modes around $k_{x}∼0$, we observe an interesting difference. In Figure 1g,h, the emission from the metasurface close to normal incidence is plotted. In these plots, four different modes are visible. The modes labeled by $p_{y}$ and $p_{x}$ are bright for all wave vectors and correspond to two dipolar modes along the y‐ and x‐directions, respectively. Both modes are similar for the Si and Ag arrays. At higher energies there are two symmetry‐protected BICs. Due to the vanishing of the overlap of the mode profiles with the free‐space radiation at $k_{x}=0$, their extinction approaches zero at this wave vector. For the Si arrays, we showed in a recent article [30] that they correspond to magnetic dipolar (MD) mode in the *z* direction $\left(m_{z}\right)$, and an electric quadrupolar (EQ) mode in the *xy*‐plane $\left(Q_{xy}\right)$, as shown in Figure 1g. In the Si metasurface, there is a splitting between the magnetic and quadrupolar modes of approximately 10 meV. In contrast, these modes are (almost) degenerate in the Ag metasurface resulting in hybrid multipolar modes ($m_{z}\pm i{\tilde{Q}}_{xy}$, indicated in Figure 1h), as will be explained in more detail below and in Supporting Information S1: Section S4, where the far field polarization of the different modes is shown.

To shed light on the underlying physics, we carried out numerical calculations using the free software SMUTHI (scattering by multiple particles in thin‐film systems). The details on the simulations and simulated dispersions can be found in Supporting Information S1: Section S5 of the SI [43]. SMUTHI is based on the T‐matrix method to account for the single particle scattering and on the scattering‐matrix method for the propagation through a layered medium. The dipolar/quadrupolar nature of the modes is confirmed with SMUTHI by calculating extinction bands up to the dipolar (l=1), quadrupolar (l=2), and octupolar (l=3) order, the latter only to discard higher‐order contributions. The condensation mode in the case of the Si metasurface is attributed to the EQ BIC, spectrally separated from all the MD modes. This separation in the case of Si appears because the fields of the magnetic mode have a larger contribution inside the particle compared to the Ag array, whereas the fields of the quadrupolar lie mostly outside the particle for both Si and Ag metasurfaces (Supporting Information S1: Section S6). In the case of the Ag metasurface, the calculations confirm the spectral overlap of the EQ and MD modes. Remarkably, SMUTHI allows for the identification of two related BICs separated by $∼10^{-3}$ eV, due to the fact that the multipoles of cylinders are very accurately accounted for. In practice, both modes ought to be mixed due to the obvious imperfections of the fabricated nanodisks. This will be further discussed below in connection with the condensate vortex emission shape and polarization.

When excitons are generated with sufficient laser power (λ=400 nm, 1 kHz, 100 fs pulse from Astrella, Coherent), they can relax into a single mode, forming a nonequilibrium polariton condensate or polariton laser. The increase of the emission from the BIC upon reaching the condensation threshold is very similar for the Ag and Si arrays, as shown in Figure 2a, where the emission intensity at $k_{x}=0.05\;\mu m^{-1}$ is plotted as a function of the absorbed fluence. The absorbed fluence was calculated from the incidence power, measured with a Newport 1918 818‐UV head power meter, multiplied by the absorbed fraction of the laser light at 400 nm (0.15), the transmission of the overfilled objective lens (0.25), Nikon Plan Fluor ELWD 40x 0.6NA, and divided by the area of the excitation spot (Gaussian spot with FWHM of 120 μm) measured from the PL below threshold by the real space camera. Both systems also show a similar narrowing of the emission linewidth upon reaching threshold at a pump fluence around 14 μJ ${\text{cm}}^{-2}$, resulting in a linewidth of ∼0.4 nm for a excitation spot size with a diameter of 120 μm.

**FIGURE 2:**
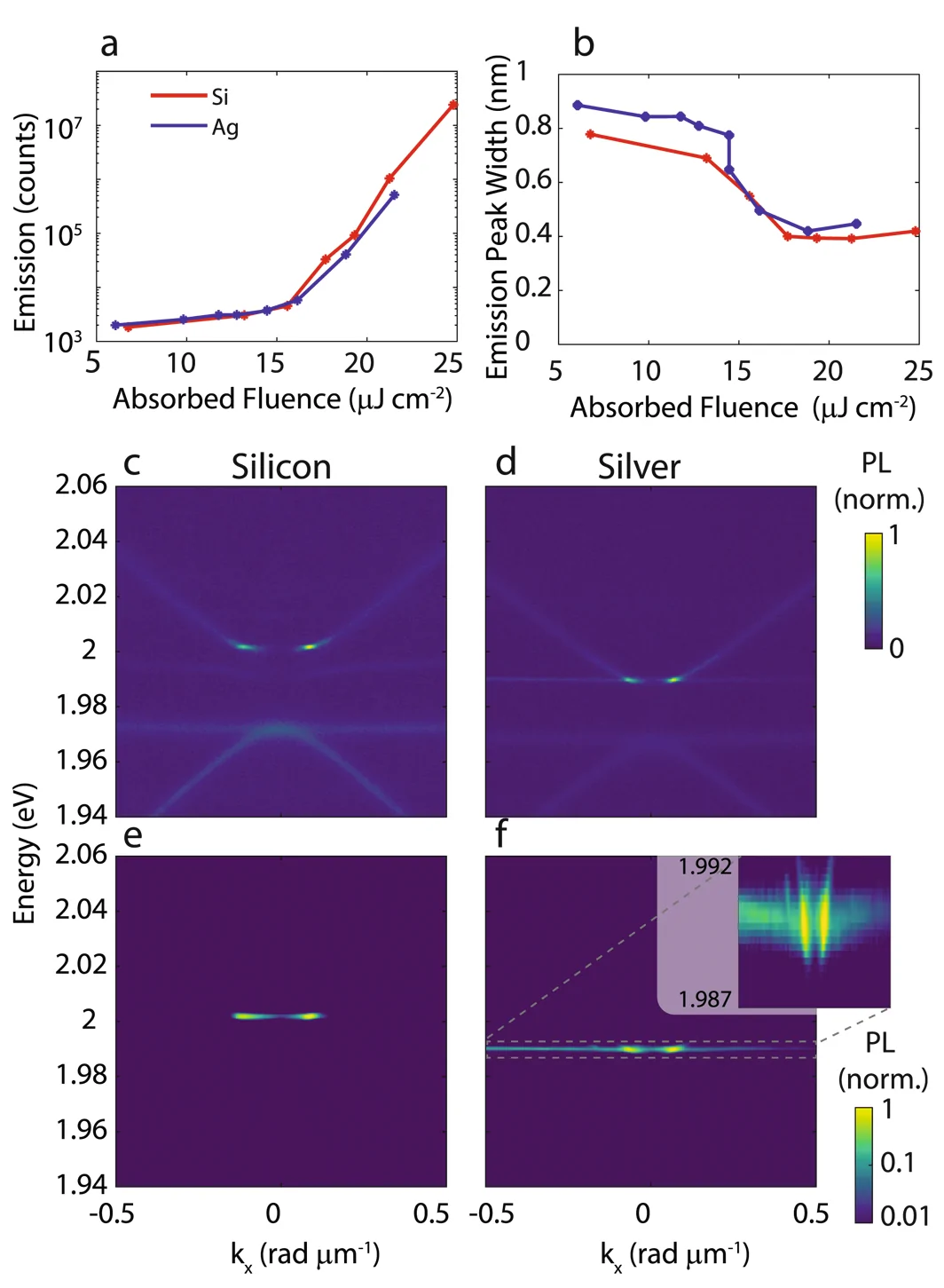
(a) Condensation threshold curves for the Si (red curve) and Ag (blue curve) metasurfaces. (b) Linewidths of the emission from the condensates at $k_{x}=0.05$ rad $\mu m^{-1}$ as a function of pump fluence for Si (blue curve) and Ag (red curve). (c) and (d) Emission dispersion at threshold (*p* = $P_{th}$) for the Si and Ag metasurfaces, respectively. (e) and (f) Emission dispersion at *p* ∼1.3$P_{th}$ on a logarithmic color scale. The dispersions are plotted as a function of $k_{x}$ with $k_{y}$ = 0.

In the Si metasurface, the excitons relax into the quadrupolar mode, as observed in the dispersion at the condensation threshold ($P_{th}$) in Figure 2c. In this figure, the polaritons that have started to condense into the BIC are leaking from the condensate at two discrete points in reciprocal space. The condensation takes place into the quadrupolar BIC, due to its longer lifetime compared to the lifetime of the magnetic BIC, which enables a faster buildup of the polariton condensate before the polaritons decay (Supporting Information S1: Figure S6g and Ref. [42]). The condensate emission at the threshold in the Ag metasurface appears very similar, as shown in the emission at two discrete wave vectors in Figure 2d. The main differences are that the emission is at slightly lower energy and smaller wave vectors compared to the Si array, due to the different coupling between the lattice mode and the localized resonances of the particles.

Due to the near degeneracy of the two BICs, a pronounced difference between the Si and Ag metasurfaces becomes apparent when the emission above threshold (*P* = 1.3 $P_{th}$) is compared. To emphasize this difference, we plot the emission on a logarithmic scale for the Si and Ag metasurfaces in Figure 2e,f, respectively. In addition to the emission at the two discrete wave vectors, the condensate in the Ag metasurface also emits from a quasi‐flat band that follows the dispersion of the hybrid multipolar BIC $\left(m_{z}+i{\tilde{Q}}_{xy}\right)$, as shown in the inset of Figure 2f. Although this mode is also a BIC, a nonzero emission is visible at $k_{x}$ = 0, which is explained by the width of the spectrometer slit, collecting emission from wave vectors slightly larger than $k_{y}$ = 0, where the mode is not dark. The intensity of the emission from this flat band at $k_{y}$ = 0 is relatively low. However, at slightly larger values of $k_{y}$ its emission dominates, as discussed below in Figure 3 and in Supporting Information S1: Section S7.

**FIGURE 3:**
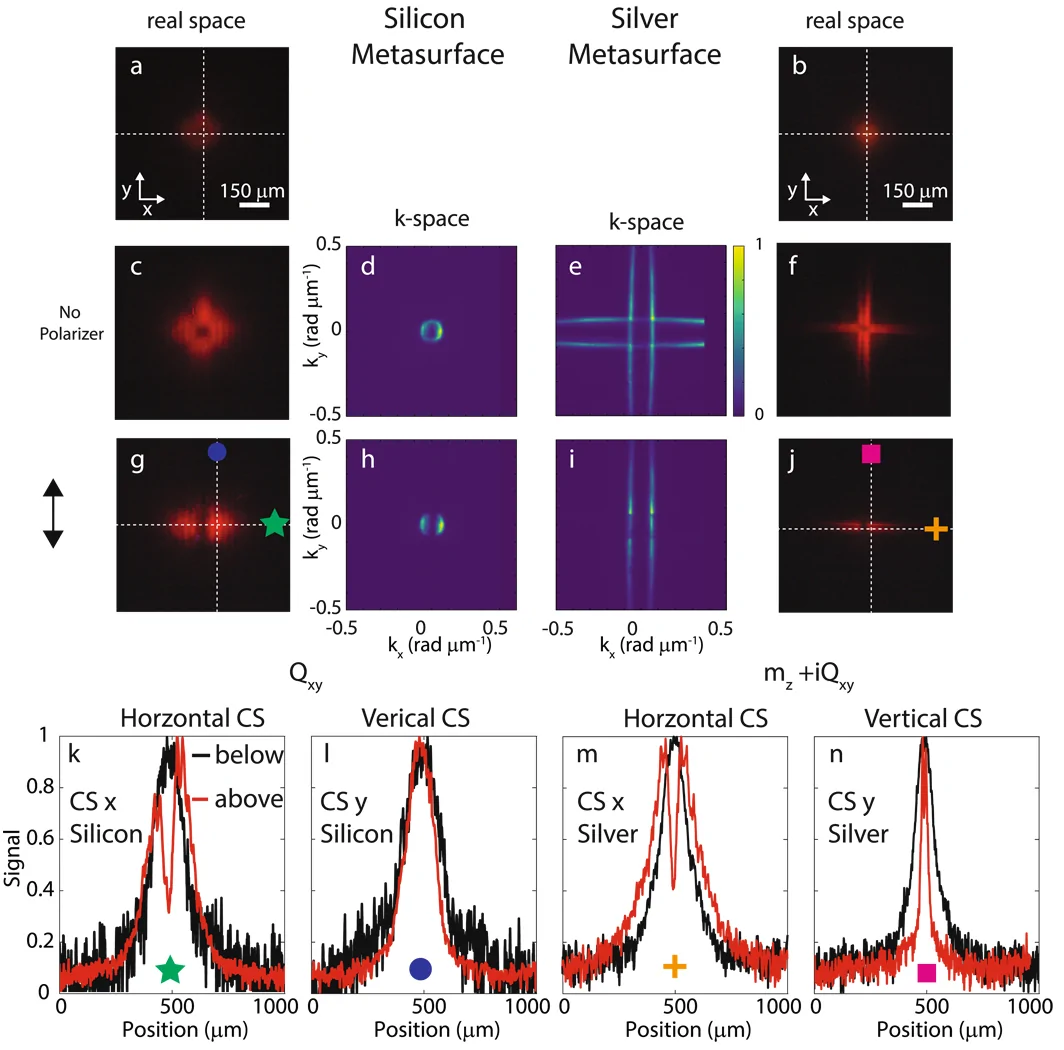
To fully investigate the condensation mode of the Ag and Si arrays, we map the condensate in both real space (red plots) and reciprocal space (green/blue plots) using different orientations of the polarizer in the detection. (a) and (b) show the area of emission below threshold. (c–f) show the emission from the condensate without a polarizer. (g–j) show the vertical polarized fraction of the emission. The emission for the other polarizations are plotted in the Supporting Information S1: Figure S11. The cross sections of the real‐space dimensions of the condensate (along the white dashed lines) are plotted in (k–n) and the resulting widths corresponding to the confinement of the condensates are given in Table 1. The contrast has been enhanced in (a) for better visibility of the spot.

Next, we examine the effects of the flat bands on spatial confinement of the condensates. In 2D metasurfaces, the group velocity is given by $v_{g}=\nabla_{k}\omega$ with k = $\left(k_{x},k_{y}\right)$. The group velocity along the *x*‐axis of the lattice is therefore $v_{g_{x}}=\frac{d\omega }{dk_{x}}=\hbar \frac{dE}{dk_{X}}$ and can thus be calculated from the dispersion of the mode in the $k_{x}$ versus energy plane. The appearance of the flat band in Figure 2f thus implies a low group velocity of the polaritons along the *x*‐direction, which is confirmed when we calculate the group velocities of the different modes from their dispersion: We fit the peaks of the dispersion of the magnetic and quadrupolar modes in the Si array in Figure 4a, and those of the hybrid $\left(m_{z}+i{\tilde{Q}}_{xy}\right)$ and $\left(m_{z}-i{\tilde{Q}}_{xy}\right)$ modes in the Ag array in Figure 4b, where the maxima are indicated with the white circles. From the resulting dispersion, we obtain the group velocities of the modes from the derivative. Because noise in the peak position is amplified when taking the derivative, the peak positions are smoothed by a moving average, as shown with the white curves in Figure 4a,b. The group velocity of the hybrid $\left(m_{z}+i{\tilde{Q}}_{xy}\right)$ mode in the Ag array (red dashed curve in Figure 4c) is clearly lower (along $k_{x}$) than the group velocity of the quadrupolar ($Q_{xy}$ mode) in Si (blue solid curve in Figure 4c). At $k_{x}$ = 0.2 rad $\mu m^{-1}$, the group velocities for the Ag array are 0.45 c, where c is the speed of light in vacuum, for ($m_{z}-i{\tilde{Q}}_{xy}$), and 0.012 c for ($m_{z}+i{\tilde{Q}}_{xy}$), whereas for the Si array, the group velocities are 0.39 c for the $Q_{xy}$ mode and 0.08 for the $m_{z}$ mode (blue dashed curve in Figure 4c). Therefore, at this wave vector, the group velocity of the quasi‐flat band in the Ag array is almost 40 times slower than the quadrupolar mode in the Si array, implying a much stronger localization of the polariton condensate in quasi‐flat band of the Ag metasurface (a detailed analysis of the vectorial group velocity throughout the entire reciprocal space is presented in Supporting Information S1: Section S9). As discussed in ref. [30], the ballistic propagation of condensates away from the excitation region, leads to an increase in the threshold. Therefore, the reduced group velocity in the quasi‐flat band of the Ag array compared to the Si array can partially explain the similar condensation thresholds in Ag and Si metasurfaces, despite the larger materials losses in Ag. We note that a critical parameter defining the condensation threshold is the energy difference between the exciton reservoir and the lower polariton. This energy difference is more relevant than the excitonic fraction, as it allows efficient relaxation pathways through radiative pumping and vibronic‐assisted scattering [44]. For the comparison between Si and Ag arrays, we have tuned the arrays' dimensions to obtain the lower polaritons at the same energies.

**FIGURE 4:**
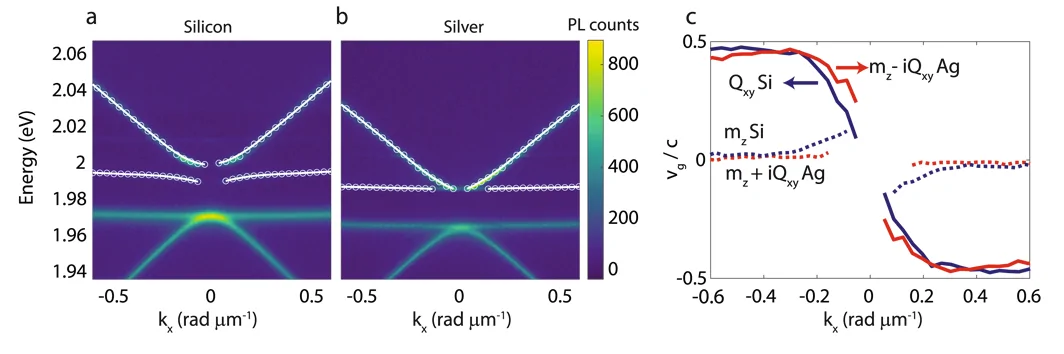
The dispersion of the Si array (a) and Ag array (b) with the peak maxima of the (quasi) BICs indicated with the white circles. The white curves are a smoothed curve through the data used to calculate the derivative. (c) Group velocity of the magnetic dipolar and electric quadrupolar modes in the Si array represented by the blue solid and dashed curves, respectively, and the group velocity of the hybrid modes $(\left(m_{z}+i{\tilde{Q}}_{xy}\right)$ and $\left(m_{z}-i{\tilde{Q}}_{xy}\right))$ in the Ag array by the red solid and dashed curves, respectively.

To further study the condensates, we have measured the emission in Fourier and real space. Because the emission profiles in real and Fourier space are related through the Fourier transform, the extent of the emission Δk (the full width at half maximum of the peaks) in Fourier space is inversely proportional to the propagation length of the polaritons $L_{p}\propto \frac{1}{\Delta k}$ (derivation in Supporting Information S1: Section S8). Therefore the localization should be higher for the modes with a larger spread in Fourier space. To verify this, we excite the metasurfaces with a Gaussian beam, which defines the shape of the emission at low excitation fluences. Below threshold, the fluorescence is dominated by incoherent polaritons, as shown in Figure 3a,b for the Si and Ag metasurfaces, respectively. When the threshold is reached, the emission profile becomes dominated by the coherent emission from the condensate, leading to very different properties. For the Si metasurface, the fluorescence (real space) originates from a donut shape area with four lobes (Figure 3c), and the far‐field emission measured in the Fourier plane resembles a vortex with no emission in the normal direction due to the quadrupolar BIC (Figure 3d) [29]. The emission from the condensate in the Ag metasurface is very different. The far‐field emission shows a double cross that spreads over much larger wave vectors (Figure 3e). The flat band that was observed in the dispersion in Figure 2f, agrees with these extended bands. Due to the distribution of the condensate over a wide range of wave vectors and the slow group velocity, we would expect a confined condensate in real space. However, this confinement is not directly clear from the real space image of the condensate, resembling an extended cross shape as shown in Figure 3f.

To explain this last observation, we have added a polarizer in the detection path to distinguish between the different polarizations of the condensate. When the polarizer is oriented along the *y*‐direction, the emission from the Si metasurface only originates from two lobes along the *x*‐direction (Figure 3g). For this polarization, there is no emission at $k_{y}=0$ (Figure 2h), which is in agreement with the polarization of an electric quadrupole vortex. For the Ag metasurface, the emission originates only from the bands parallel to the $k_{y}$ axis (Figure 3i). The *y*‐polarized emission has very confined momentum along $k_{x}$, resulting in a low spatial confinement along this direction (Figure 3j). However, the momentum of the condensate takes a broad range of values along $k_{y}$, resulting in the spatial confinement of the condensate along the *y*‐direction. This explains why, without a polarizer, the condensate does not appear spatially confined. A further analysis of the group velocities of all modes is shown in the Supporting Information S1: Section S9.

To compare the confinement of the condensates, we take the profile of the excitation spot and the condensates in real space, along the white dashed lines in Figure 3a,b,g, and j. The cross sections are shown in Figure 3k along the *x*‐axis below threshold (black curve), and above threshold (red curve) for *y*‐polarized emission. Although the shape of the emission below and above threshold are very different, making a quantitative comparison difficult, we can still observe some clear trends. Upon condensation, the shape of the emission changes into two peaks, with a total width slightly larger than that below threshold (the FWHM, directly obtained from the width of the peaks at 50% of the maximum value, is ∼215μm below threshold and ∼290μm above threshold for the two peaks together). However, the individual peaks have a width of approximately one third of this value, that is ∼100μm. Along the *y*‐direction, the spot is actually a bit smaller above threshold than below, reducing from ∼215μm to ∼160μm, as shown in Figure 3l. This reduction can be understood by the nonlinear dependence of the condensate population with the exciton density. It is therefore much more efficient to form the condensate in the center of the excitation region, where this density is larger than at the edges. Above the threshold, the emission is dominated by the condensate, and the overall width reduces.

For the Ag metasurface, the expansion of the condensate above threshold along the *x*‐direction is even larger than for the Si array, increasing the FWHM from ∼160μm below threshold to ∼250μm above threshold (see Figure 3m). This expansion is not surprising because the Fourier image shows the emission at a well‐defined wave vector along the *x‐*direction (Figure 3i), implying larger uncertainty in the position. The emission profiles for the Ag array along the *y*‐direction are more interesting. We see that above the threshold the size sharply decreases from ∼160μm to ∼50μm (see Figure 3n). The nonlinear process of condensation, combined with the excellent spatial confinement, explains this strong contraction of the emission profile. The excellent confinement is explained by the low group velocity associated with the flat band and broad distribution of the condensate's wave vectors along $k_{y}$. An overview of the condensate and excitation profiles is given in Table 1. These results agree with the group velocities of the condensates in the *x*‐ and *y*‐directions for the Ag and Si metasurfaces, respectively, as discussed in Figure 4.

**TABLE 1:** Excitation diameter, condensate size for vertical polarization, and ratio for Si and Ag cavities.

	Excitation diameter	Condensate size for vertical polarization	ratio (condensate/excitation)
Si			
*x*	215 μm	290 μm	1.35
*y*	215 μm	160 μm	0.74
Ag			
*x*	160 μm	250 μm	1.56
*y*	160 μm	50 μm	0.31

We have further analyzed the condensation mode using COMSOL Multiphysics, calculating the multipolar decomposition of the BIC mode near the Γ point. Figure 5a shows the contributions of two nondegenerate modes for the Ag array with different multipolar behavior: a dipolar magnetic contribution along the *z*‐axis, $m_{z}$, and an electric quadrupole in the xy−plane, $Q_{xy}$. These two modes also appear in the Si arrays, but in that case, they are clearly separated in frequency. To directly compare $Q_{xy}$ with $m_{z}$, we defined the magnitude ${\tilde{Q}}_{xy}=\frac{\omega }{6}Q_{xy}$, which has units of magnetic moment. As shown in Figure 5a, both modes overlap and are dispersive, and the decomposition does not reproduce the experimental results. Therefore, we need to find base that reproduces the different dispersion of the two modes.

**FIGURE 5:**
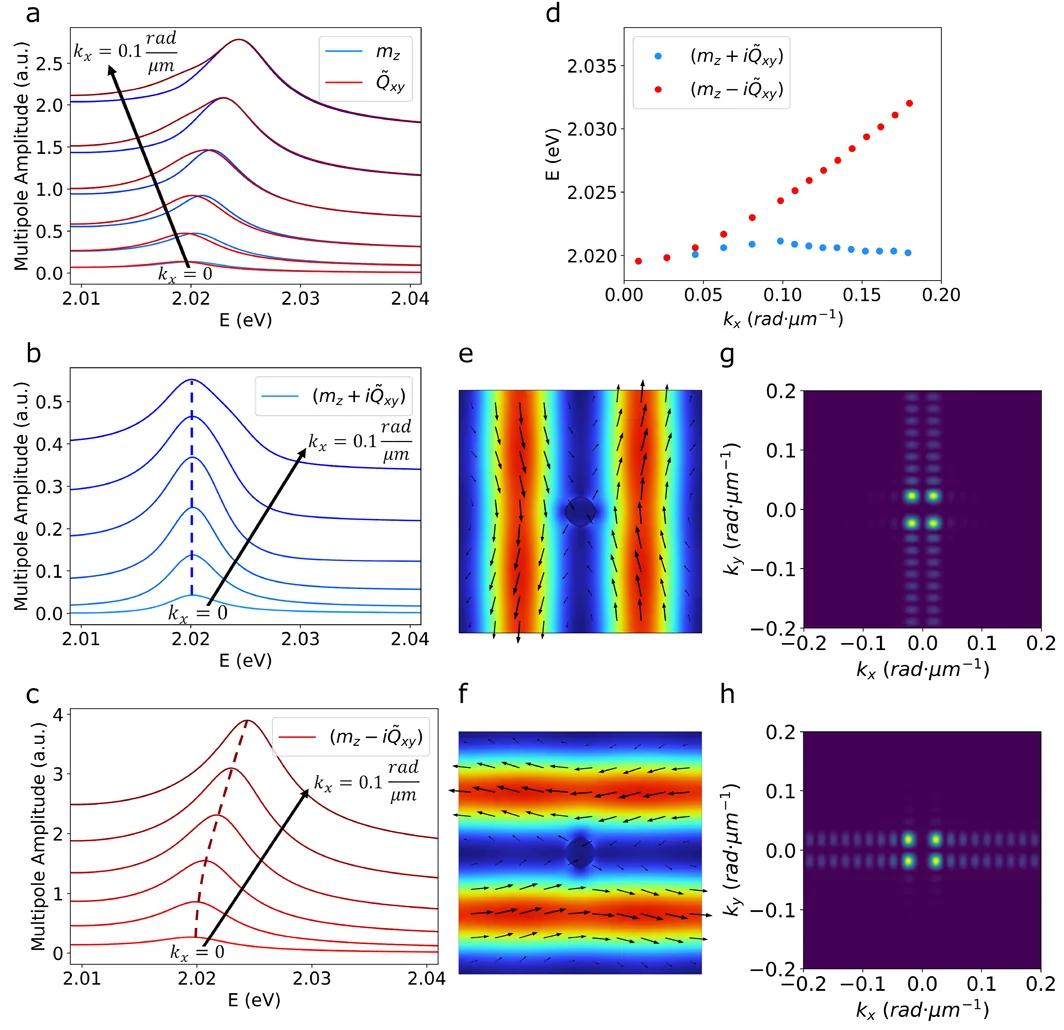
(a) Multipolar decomposition of the BICs in the Ag array obtained from the scattering simulations for different values of the in‐plane wavevector $k_{x}$. The plot shows the contributions of a magnetic dipole along the *z*‐axis, $m_{z}$ (blue curves), and an electric quadrupole in the xy−plane, ${\tilde{Q}}_{xy}$ (red curves). The modes are not independent, and the decomposition is not appropriate. (b) and (c) Multipolar decomposition of the BICs in the Ag array using the $\{\left(m_{z}+i{\tilde{Q}}_{xy}\right)$, $\left(m_{z}-i{\tilde{Q}}_{xy}\right)\}$ basis. The two modes are now clearly separated, with the $\left(m_{z}+i{\tilde{Q}}_{xy}\right)$ mode accounting for the flat band. (d) Energy dispersion of the $\left(m_{z}+i{\tilde{Q}}_{xy}\right)$ (blue dots) and $\left(m_{z}-i{\tilde{Q}}_{xy}\right)$ modes (red dots) as a function of the in‐plane wavevector $k_{x}$. Near field distributions of the $\left(m_{z}+i{\tilde{Q}}_{xy}\right)$ (e) and $\left(m_{z}-i{\tilde{Q}}_{xy}\right)$ (f) modes, obtained from eigenmode analysis. Theoretical far‐field emission of the $\left(m_{z}+i{\tilde{Q}}_{xy}\right)$ (g) and $\left(m_{z}-i{\tilde{Q}}_{xy}\right)$ (h) modes for an array with *N* = 750 unit cells according to Equation (3). Note that the y‐polarized emission from panel (g) matches well with the experimental emission from Figure 3i.

The radiation fields, ${\vec{E}}_{m}$ and ${\vec{E}}_{Q}$, of a magnetic dipole $\vec{m}$ and an electric quadrupole $\overset{↔}{Q}$ are, respectively [45], given as follows:

(1) $${\vec{E}}_{m}=-\frac{Z_{0}k^{2}}{4\pi }\frac{e^{\mathrm{ikr}}}{r}\left(\hat{r}\times \vec{m}\right),\;{\vec{E}}_{Q}=\frac{ik^{3}}{24\pi \varepsilon_{0}}\frac{e^{\mathrm{ikr}}}{r}\left[\left(\hat{r}\times \vec{Q}\left(\hat{r}\right)\right)\times \hat{r}\right],$$

where $\hat{r}$ is the unit vector of the direction of observation, $Z_{0}=\sqrt{\mu_{0}c}$ is the impedance of free space, $\vec{Q}\left(\hat{r}\right)=\overset{↔}{Q}\cdot \hat{r}$ is the projection of the electric quadrupole tensor along $\hat{r}$, and the harmonic time dependence is implicit. If we consider the components of the relevant multipoles, $m_{z}$ and $Q_{xy}$ in this case, these relations can be further simplified to the following equation:

(2) $${\vec{E}}_{m_{z}}=-\frac{Z_{0}k^{2}}{4\pi }\frac{e^{\mathrm{ikr}}}{r}m_{z}\left(\begin{array}{c} r_{y}\\ -r_{x}\\ 0 \end{array}\right),\;{\vec{E}}_{Q_{xy}}=\frac{ik^{3}}{24\pi \varepsilon_{0}}\frac{e^{\mathrm{ikr}}}{r}Q_{xy}\left(\begin{array}{c} r_{y}\left(1-2r_{x}^{2}\right)\\ r_{x}\left(1-2r_{y}^{2}\right)\\ -r_{x}r_{y}r_{z} \end{array}\right)\approx \frac{ik^{3}}{24\pi \varepsilon_{0}}\frac{e^{\mathrm{ikr}}}{r}Q_{xy}\left(\begin{array}{c} r_{y}\\ r_{x}\\ 0 \end{array}\right),$$

where $r_{i}$ are the cartesian components of $\hat{r}$, and the last approximation is valid for small angles, that is close to the Γ point, which is the region of interest. These two equations result in the far‐field vortices of $m_{z}$ and $Q_{xy}$. By looking at the prefactors of both equations and using ${\tilde{Q}}_{xy}=\frac{\omega }{6}Q_{xy}$, the total radiation field can be written as follows:

(3) $$\vec{E}={\vec{E}}_{m_{z}}+{\vec{E}}_{Q_{xy}}=-\frac{Z_{0}k^{2}}{4\pi }\frac{e^{\mathrm{ikr}}}{r}\left(\begin{array}{c} \left(m_{z}-i{\tilde{Q}}_{xy}\right)r_{y}\\ -\left(m_{z}+i{\tilde{Q}}_{xy}\right)r_{x}\\ 0 \end{array}\right).$$

According to this derivation, instead of using the modes {$m_{z}$, ${\tilde{Q}}_{xy}$} as a base, we can use a linear combination of these states: $\{\left(m_{z}+i{\tilde{Q}}_{xy}\right)$, $\left(m_{z}-i{\tilde{Q}}_{xy}\right)\}$. The results of the multipolar decomposition in the new basis are shown in Figure 5b,c. Within this picture, the two orthogonal modes become clearly independent, with $\left(m_{z}+i{\tilde{Q}}_{xy}\right)$ (Figure 5b) corresponding to the nondispersive flat band, and $\left(m_{z}-i{\tilde{Q}}_{xy}\right)$ (Figure 5c and Supporting Information S1: Section S10) corresponding to the mode with a parabolic dispersion. The energy dispersion of both modes as a function of the in‐plane wavevector $k_{x}$ is presented in Figure 5c. Figure 5e,f show the near‐field distribution of both modes at the Γ point on a plane crossing the Ag nanodisk at its center. Finally, Figure 5g,h show the theoretical far‐field radiation computed from Equation (3), which matches well with the measurements of Figure 3i and together they reproduce the double cross pattern. In summary, the emergence of the double cross pattern in Ag nanodisks arrays is allowed by the very close proximity of the bands to the in‐plane diffraction order (Rayleigh anomaly), thus yielding a *k*‐space pattern strongly governed by the symmetry of the square array. In addition, both COMSOL and SMUTHI calculations proved the presence of two (quasi‐)degenerate modes with significant dipolar and quadrupolar contributions. In addition, the features of the Ag condensate vortices for different polarizations, shown in Supporting Information S1: Figure S12, are not consistent with a square array of only vertical magnetic dipoles; rather, a quadrupolar contribution is needed to match the resulting polarizations. Finally, the simple model proposed here, making use of the multipole amplitudes provided by COMSOL, explains the shape and polarization of the observed (quasi‐)flat bands.

## 3 Conclusions

We have designed two similar square nanoparticle arrays made of either dielectric Si nanodisks or plasmonic Ag nanodisks and covered with a dye‐doped layer. Both arrays supported an electric quadrupolar $\left(Q_{xy}\right)$ and a magnetic dipolar $\left(m_{z}\right)$ BIC, at *k* = 0 where condensation occurs. In addition, due to the different distribution of the near fields, the two BICs were split by ∼10 meV in the Si array, whereas being practically degenerate in the Ag array. Simulations showed that the interference of $Q_{xy}$ and $m_{z}$ in the Ag array leads to two possible hybrid modes, $\left(m_{z}+i{\tilde{Q}}_{xy}\right)$ and $\left(m_{z}-i{\tilde{Q}}_{xy}\right)$, polarized along either the y or x axis, respectively. For the Si array, we showed that condensation takes place into the $Q_{xy}$ BIC, with its expected vortex in the far‐field emission. On the other hand, the Ag array condensed into the hybrid $\left(m_{z}+i{\tilde{Q}}_{xy}\right)$ and $\left(m_{z}-i{\tilde{Q}}_{xy}\right)$ orthogonally polarized modes.

Surprisingly, both arrays show similar condensation thresholds, regardless of the larger inherent material losses of the plasmonic Ag compared to the dielectric Si nanodisks. We speculate that these larger material losses in the Ag array are compensated for by the fact that condensation in the Ag array occurs in a quasi‐flat band with larger confinement along one direction, resulting in lower losses due to propagation in that direction, which is in agreement with recent results on the strong dependence of the condensation threshold on the polariton's dispersion and group velocity [30]. Furthermore, the larger material losses of Ag compared to Si, may play a limited role because the highest field enhancements near the BIC frequency are located outside the particle (as is visible in Supporting Information S1: Section S6).

The far‐field emission exhibited a double cross‐shaped pattern, extending over much larger wave vectors in the far field. This lack of confinement of the wave vectors was caused by the (partially) flat band nature of the $\left(m_{z}+i{\tilde{Q}}_{xy}\right)$ mode. Conversely, the spatial confinement of the condensate in the Ag array along the polarization direction was highly effective, yielding a condensate size five times smaller along the confined axis under symmetric Gaussian excitation. This method of condensate confinement could prove valuable for investigating interactions between multiple condensates, with potential applications in quantum computing.

## Author Contributions

**Anton Matthijs Berghuis:** conceptualization, methodology, investigation, writing – original draft, writing – review and editing, supervision. **Jose Luis Pura:** writing – original draft, writing – review and editing, methodology, investigation. **Rafael P. Argante:** methodology, investigation, writing – review and editing. **Shunsuke Murai:** methodology, writing – review and editing. **José A. Sánchez‐Gil:** writing – original draft, writing – review and editing, methodology, investigation. **Jaime Gómez Rivas:** conceptualization, supervision, writing – review and editing, funding acquisition, writing – original draft.

## Conflicts of Interest

The authors declare no conflicts of interest.

## Supporting information


Supporting Information S1

## Data Availability

The datasets generated and/or analyzed during the current study are available from the corresponding author upon reasonable request.
