# Supplementary material for: Confinement of Polariton Condensates in Quasi‐Flat Band BICs in Plasmonic and Dielectric Metasurfaces
Source: Nanophotonics. 2026 Apr 20;15(8):e70092. doi: 10.1002/nap2.70092 (PMC13642605; doi:10.1002/nap2.70092)
Supplement: Supplementary file 1 [file NAP2-15-e70092-s001.pdf]

Supporting Information for "Confinement of Polariton Condensates in quasi-Flatband BIC in Plasmonic and Dielectric Metasurfaces"

Anton Matthijs Berghuis[1,2], Jose Luis Pura[4,5], Rafael P. Argante[1,2], Shunsuke Murai[3], José A. Sánchez-Gil[4], and Jaime Gómez Rivas[1,2]

[1]Department of Applied Physics and Science Education and Eindhoven Hendrik Casimir Institute, Eindhoven University of Technology,, P.O. Box 513, 5600 MB Eindhoven, The Netherlands.
[2]Institute for Complex Molecular Systems-ICMS, Eindhoven University of Technology, P.O. Box 513, 5612 AJ, Eindhoven, The Netherlands
[3]Department of Physics and Electronics, Graduate School of Engineering, Osaka Metropolitan University, 5998531, Osaka, Japan.
[4]Instituto de Estructura de la Materia (IEM-CSIC), Consejo Superior de Investigaciones Científicas, Serrano 121, 28006 Madrid, Spain.
[5]GdS-Optronlab, Física de la Materia Condensada, Universidad de Valladolid , Paseo de Belén 19, 47011 Valladolid, Spain

Contents

S1 Sample Fabrication **3**

S2 FDTD simulation of Silver and Silicon Metasurfaces **3**

S3 Fourier Microscope Design **3**

S4 Polarization resolved dispersion measurements **5**

S5 Band structure resolved full reciprocal space - Experiment and Simulations **7**
S5.1 3D Fourier Measurements 7
S5.2 SMUTHI Calculations 9

S6 Near-fields and Q-factors of the BICs **10**

S7 Flat band lasing at k_y >0 **11**

S8 Relation between propagation length and mode linewidth in Reciprocal space **12**

S9 Group velocities in full reciprocal space **14**

S10 Calculated dispersions of different multipole components in the Ag array **16**

S11 Condensation at different energies in Ag array **17**

S12 Real space and reciprocal space images for different orientations of the detection polarizer **18**

S13 Hopfield coefficients **19**

S14 Multipolar Decomposition Details **21**

S1 Sample Fabrication

The silicon metasurfaces were fabricated by growing polycrystalline Si with a thickness of 90 nm on a synthetic silica glass substrate with low-pressure chemical vapor deposition. A resist (NEB22A) was deposited on the film and exposed to electron-beam lithography (F7000S-KYT01, Advantest, Japan), followed by development to make nanoparticle arrays of resist on the Si film. Then, the films were dry etched with a Bosch process using a resist pattern as a mask. The remaining resist was etched away with oxygen dry etching.

The Ag metasurfaces were also fabricated by electron-beam lithography, but with a positive type resist (ZEP520A). After the development of the resist hole patterns, a Ti layer (3 nm) and an Ag layer (60 nm) were deposited on the resist by electron beam deposition. The Ag nanoparticles were formed by lift-off using a solvent (ST-120). The Ag arrays were coated with a 20 nm aluminum oxide layer by atomic layer deposition such that the particles do not oxidize.

S2 FDTD simulation of Silver and Silicon Metasurfaces

The different permittivities of Ag and Si result in a much larger scattering cross section and very different resonant wavelengths for Ag nanoparticles with the same size as the Si nanoparticles. The blue curve in Fig. S1 shows the extinction of a lattice of Si nanodisks with a diameter of 90 nm and a height of 90 nm on a quartz substrate (n=1.46) with a superstrate of n=1.6. This design results in a bright SLR with a Q-factor of $\sim$200. A lattice with the same particle size but made from silver would give a very different extinction, as shown with the pink curve in Fig. S1. The SLR is red-shifted by more than 50 nm, and the Q-factor is reduced to $\sim$ 12. To fabricate Ag metasurfaces with similar properties as the Si arrays, the size of the silver particles was reduced such that the bright SLR is at approximately the same energy as the Si array and has a similar Q-factor. This is the case when the Ag disks have a height of 60 nm and a diameter of 50 nm, as shown by the red curve in Fig. S1.

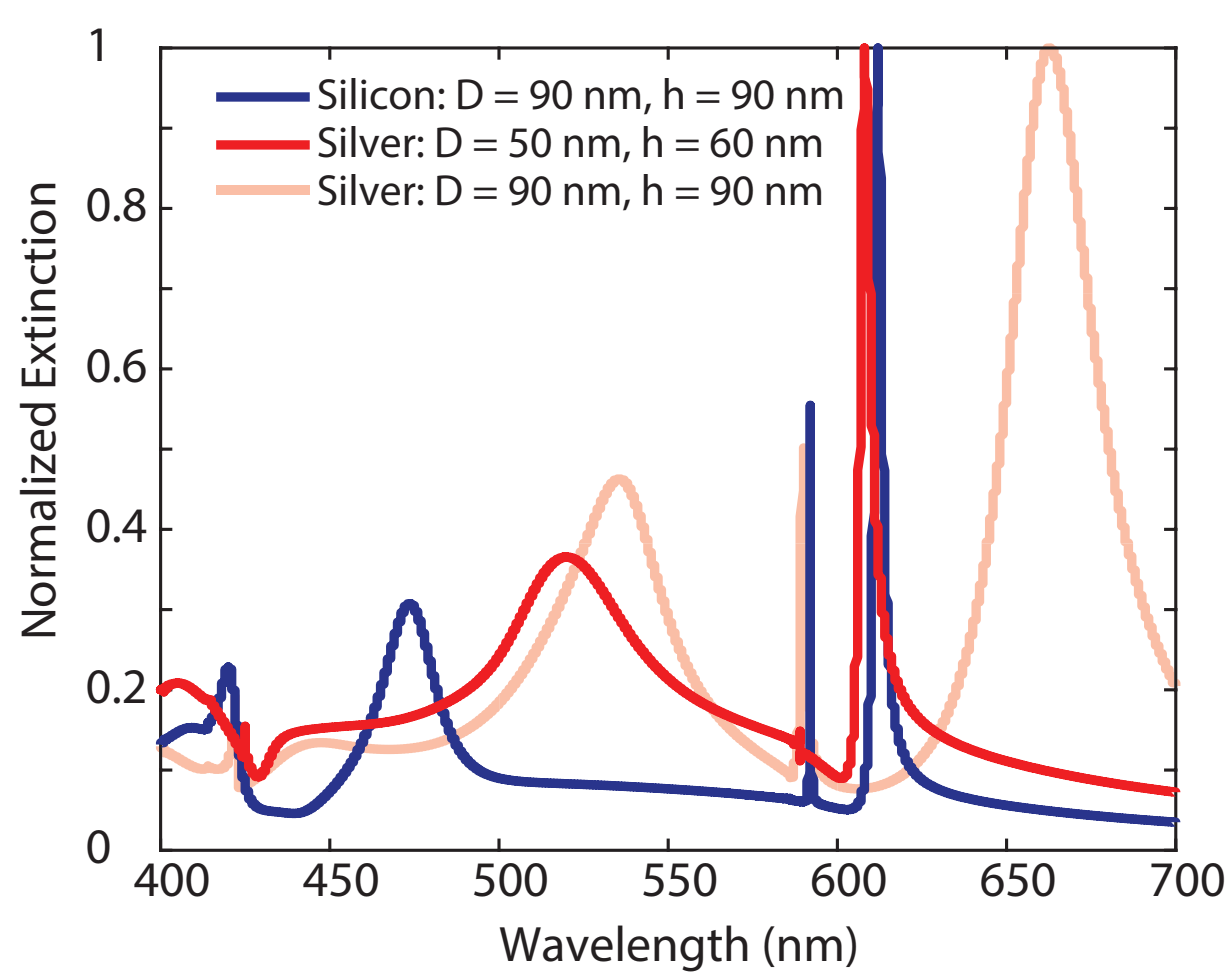

Figure S1: Extinction spectra of an array (a = 420 nm) of Si disks (D=90 nm, h=90 nm, blue curve), Ag disks (D=50 nm, h=60 nm, red curve), and Ag disks (D=90 nm, h=90 nm, pink curve).

S3 Fourier Microscope Design

To measure the energy and momentum resolved extinction of the metasufaces, we use a Fourier microscope in transmission mode. The excitation objective (40x, 0.6NA Nikon Plan Fluor ELWD)

focuses white light onto the sample. The transmission is collected by a 60x, 0.7 NA (Nikon Plan Fluor ELWD) or 10x, 0.3 NA objective. The back focal plane of this objective is imaged on the spectrometer's slit (Princeton Instruments SP2300) using 2 lenses in a 4f configuration (see Fig. S2). The spectrometer transmits light along one of the principal axes of the metasurface (k_x or k_y) and disperses it on the CCD camera (Princeton Instruments ProEM:512) using a 150 or a 600 lines/mm grating, generating an image as a function of incident angle or wave vector, and wavelength or energy. For fluorescence/condensation measurements, the objective is replaced by a lens with a focal distance f = 50 mm, and a $\lambda = 400$ nm laser at 1 kHz repetition rate is used for excitation. This laser beam is generated by frequency doubling the output of a Ti:sapphire regenerative amplifier (Coherent Astrella), $\lambda = 800$ nm with a pulse duration of 100 fs. By changing the position of the excitation lens, the excitation spot size is controlled.

To measure the 3-dimensional dispersions (k_x vs k_y vs wavelength, Figure S5), the spectrometer is placed on a motorized stage and scanned laterally. Each position allows to acquire a k_x vs wavelength for a fixed k_y value. In this way, many images are taken at different values of k_y. When these images are combined, the full 3D band structure can be reconstructed.

For real space imaging, a flipping mirror is inserted after the imaging lens, and an additional lens creates a real space image on the camera (Kiralux CS135CU, Thorlabs).

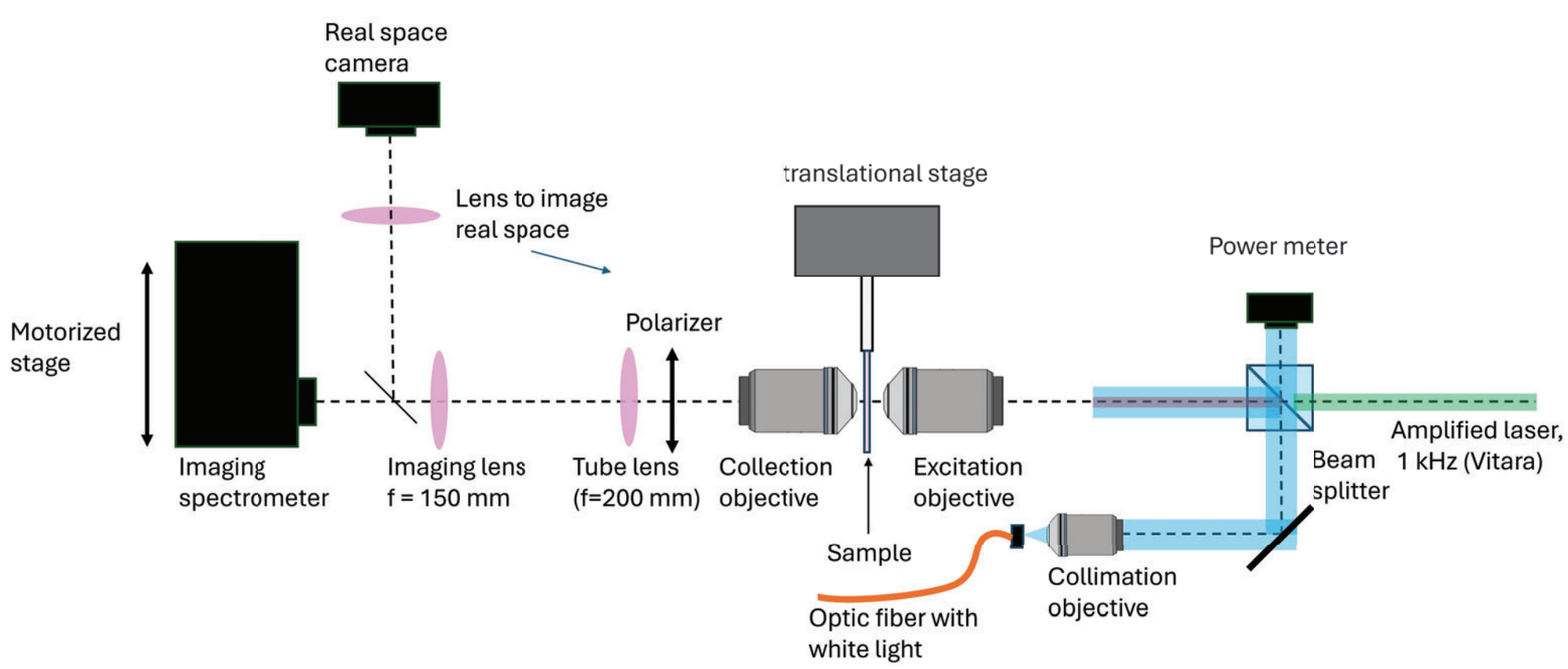

Figure S2: Schematic illustration of the Fourier Microscope

S4 Polarization resolved dispersion measurements

In the manuscript, we analyzed the dispersion measurements without polarizer. Therefore, we were not sensitive to the far-field polarization of the different modes. Here, we analyze the polarization-dependent dispersions as a function of k_x at k_y=0 and $k_y > 0$ ($k_y \sim 0.04$ rad μm^{-1}) for the Si metasurface, Fig. S3, and the Ag metasurface, Fig. S4.

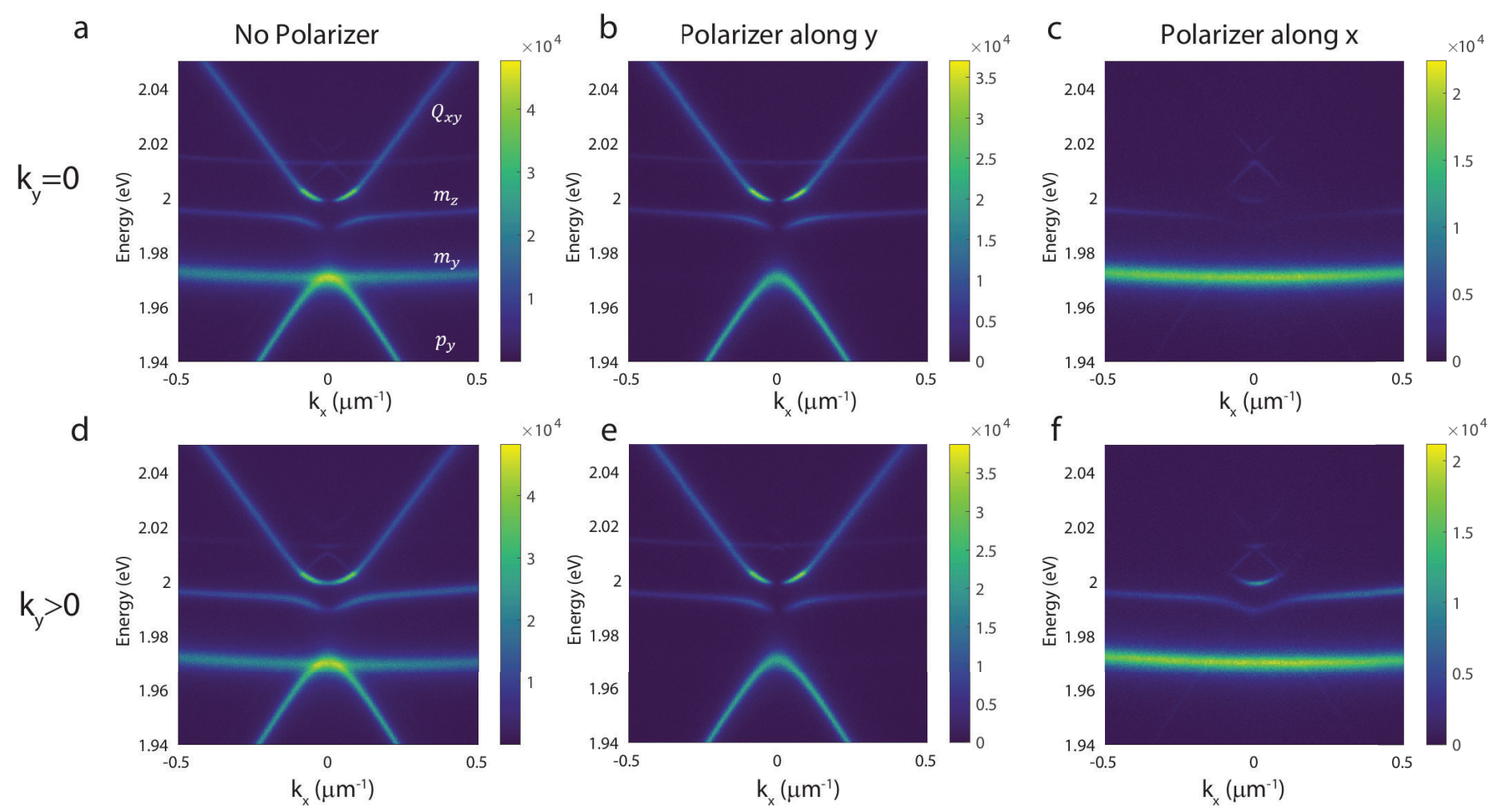

Figure S3: For the Si Array, we see for the unpolarized emission (a) two BICs, the magnetic BIC and the quadrupolar BIC. Out of normal incidence (k$_x$ >0), both quasi-BICs are bright. This is, of course, also the case for k$_y$ >0. This can be seen if we take the slice at k$_y$ >0 (panel d). We then see that both the quadrupolar and the magnetic BIC are bright, even at k$_x$=0. Interestingly, when we only look at the y polarization (b), there is no emission at k$_x$=0, not just at k$_y$=0 but also when k$_y$ >0 (e). This agrees with the polarization vortex of both m_z and Q_{xy}, which presents x-polarization for the k_y axis; thus, along k$_y$ >0 the mode becomes radiative for the x-polarization. This can be seen in Figs (c) and (f).

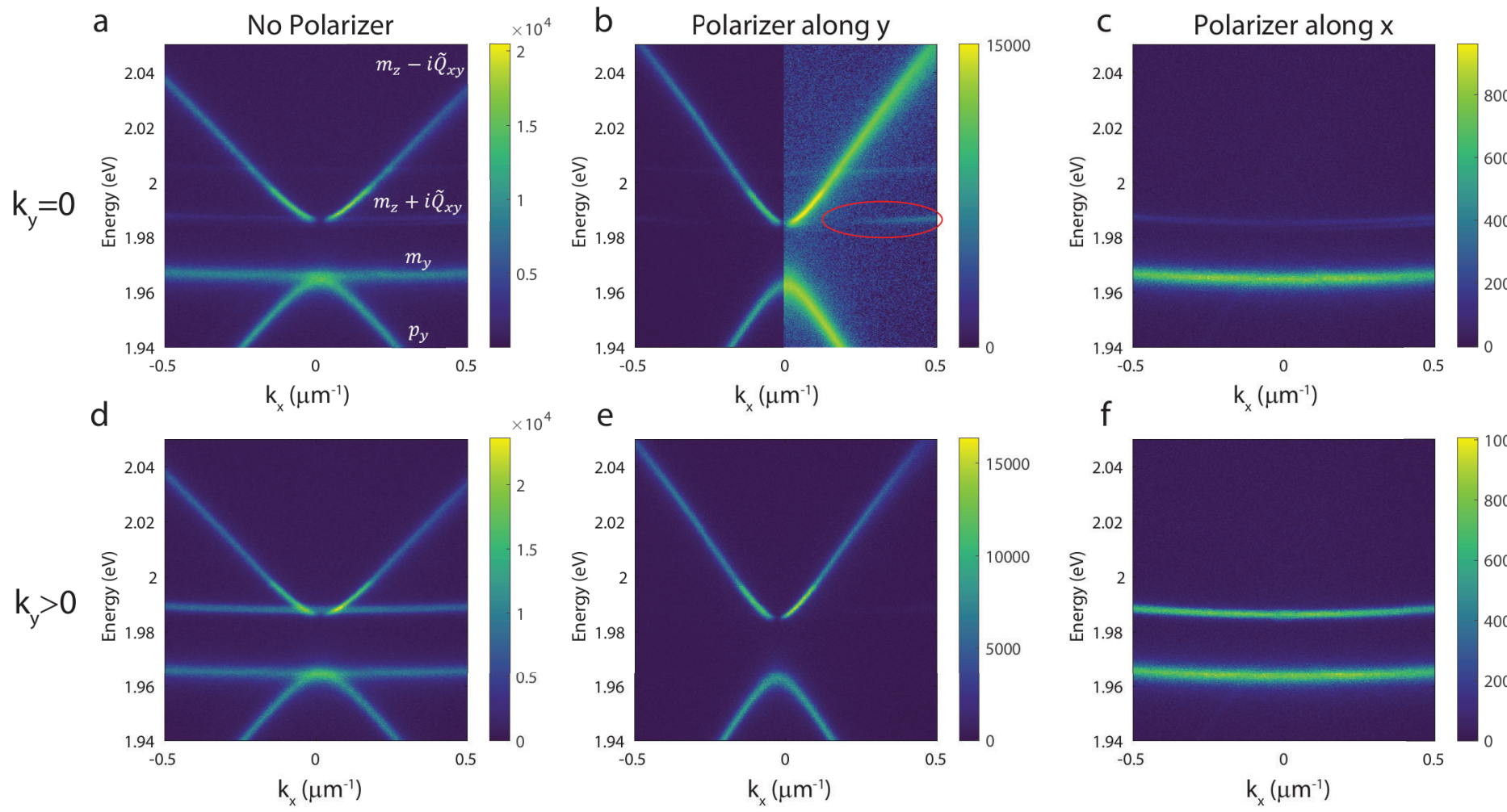

Figure S4: For the Ag array, the experiments are more difficult to understand. The reason is that the flat band $(m_z + i\tilde{Q}_{xy})$ mode is extremely faint in this Ag array (as observed in the multipolar decomposition). This $(m_z + i\tilde{Q}_{xy})$ mode is analogous to the magnetic mode in the Si array, which we observed very clearly there. There are, however, some important differences. In panel (a), we see a clearly visible flat band; however, in contrast to the magnetic mode in the Si array, this mode is polarized mostly along the x-direction. When we look in (b) at the y polarization, the mode is extremely faint, in fact, the mode is hardly visible on a linear scale, but only on a logarithmic scale, (right panel of (b)) we see a mode at large wave vectors. For the x polarization this flat mode is hardly visible (c), due to the finite width of the spectrometer slit, meaning that also some light emitted at $k_y > 0$ is collected. Something interesting happens when the cross section is taken at $k_y > 0$. We observe that the overall flat band is much brighter for every k_x in panels (d) and (f), confirming the nearly fully linear polarization of all observed modes. In this case, the picture is clearer: there are two bands, one flat and one dispersive, with orthogonal polarizations. This is in clear contrast with the Si metasurface in which both m_z and Q_{xy} presented the same polarizations. Finally, having an x-polarized flat band along k_x for $k_y > 0$, which becomes dark at $k_y = 0$, matches the observed flat bands in the k-space images, see Figure S12q. Moreover, the dispersive quasi-BIC for $k_y > 0$ is also dark at $k_x = 0$, in agreement with Figure S12i. Also note that this emission is only compatible with the reduced symmetry of the hybrid modes, C_{2v}, while it is not compatible with the original C_{4v} and $C_{\infty v}$ symmetries of the Q_{xy} and m_z modes, respectively.

S5 Band structure resolved full reciprocal space - Experiment and Simulations

In the manuscript, we analyzed the dispersion and the group velocities only in a single energy-momentum plane (i.e., E vs. k_x for k_y=0). We analyze here the 3D dispersion using both experiments and simulations to increase the understanding of the band structure and group velocity of the polaritons in all directions.

S5.1 3D Fourier Measurements

The band structure can be analyzed more rigorously by measuring it for all in-plane wave vectors around k=0. To perform the experiments, we placed the spectrometer on a translational stage, which enabled us to collect different slices of the Fourier plane with the spectrometer's slit, and disperse each slice in energy. Combining these measurements yields a 3D matrix of coordinates (k_x, k_y, λ). More details about the experimental setup can be found in the SI section S3. The resulting band structures are plotted for discrete energies in Fig. S5 a and c for the Si and Ag arrays, respectively. The splitting of the magnetic dipolar (MD) and electric quadrupolar (EQ) modes in the Si array is clearly visible in the cross sections between 2 and 2.032 eV, plotted in Fig. S5 a. This splitting leads to a momentum-isolated mode at an energy of 2 eV (just above the quadrupolar BIC). This mode is indicated with the red dashed circle in Fig. S5 a and will be the mode that the condensate will relax into when the pump fluence is increased. The magnetic BIC is visible in the same figure at 1.99 eV. In the Ag array, there is no splitting between the magnetic and quadrupolar modes, and therefore no momentum-isolated mode. As a result, both the magnetic and quadrupolar BICs coexist at the same plane at 1.986 eV in Fig. S5 c. The condensate takes place in the $(m_z + i\tilde{Q}_{xy})$ linear combination of both modes. The extended shape of the hybrid mode in the Fourier plane is very distinct from the isolated "donut" shape of the quadrupolar BIC observed in the Si array.

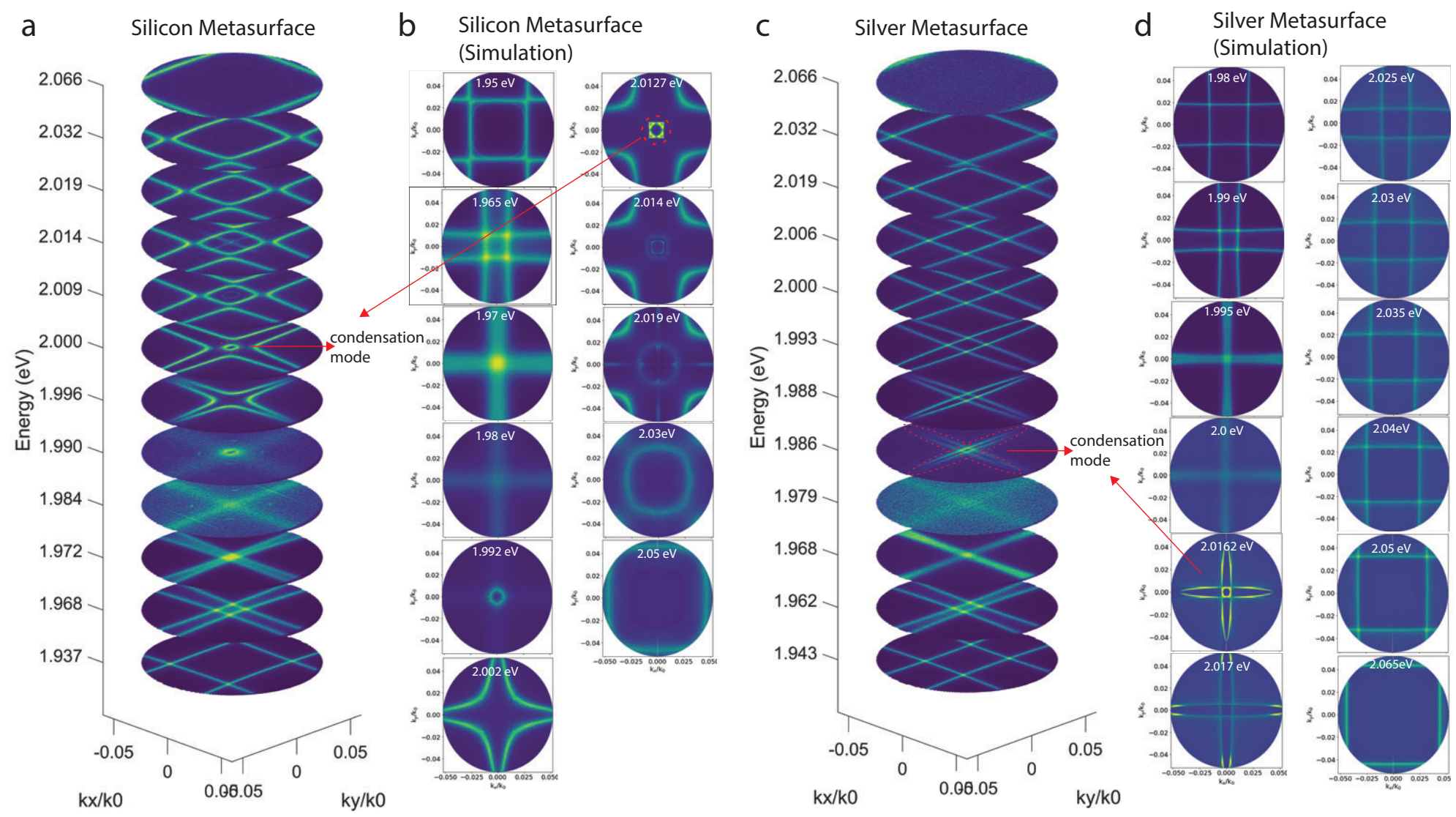

Figure S5: Isofrequency surfaces of the band structure resolved for both k_x and k_y for the Si Array (a) and for the Ag array (c) (note that the energy step between the slices is not always equal). The image is obtained by collecting the emission and is normalized for each slice. The splitting of the upper bands in the Si array is clear, resulting in quite a different dispersions for the Si and Ag arrays. This different dispersion will result in condensation into a different mode when the pump fluence is increased above the condensation threshold. The red dashed curves and arrow indicate the condensation (polariton lasing) mode. The experiments match excellently with the SMUTHI calculations, in Figs. b and d.

S5.2 SMUTHI Calculations

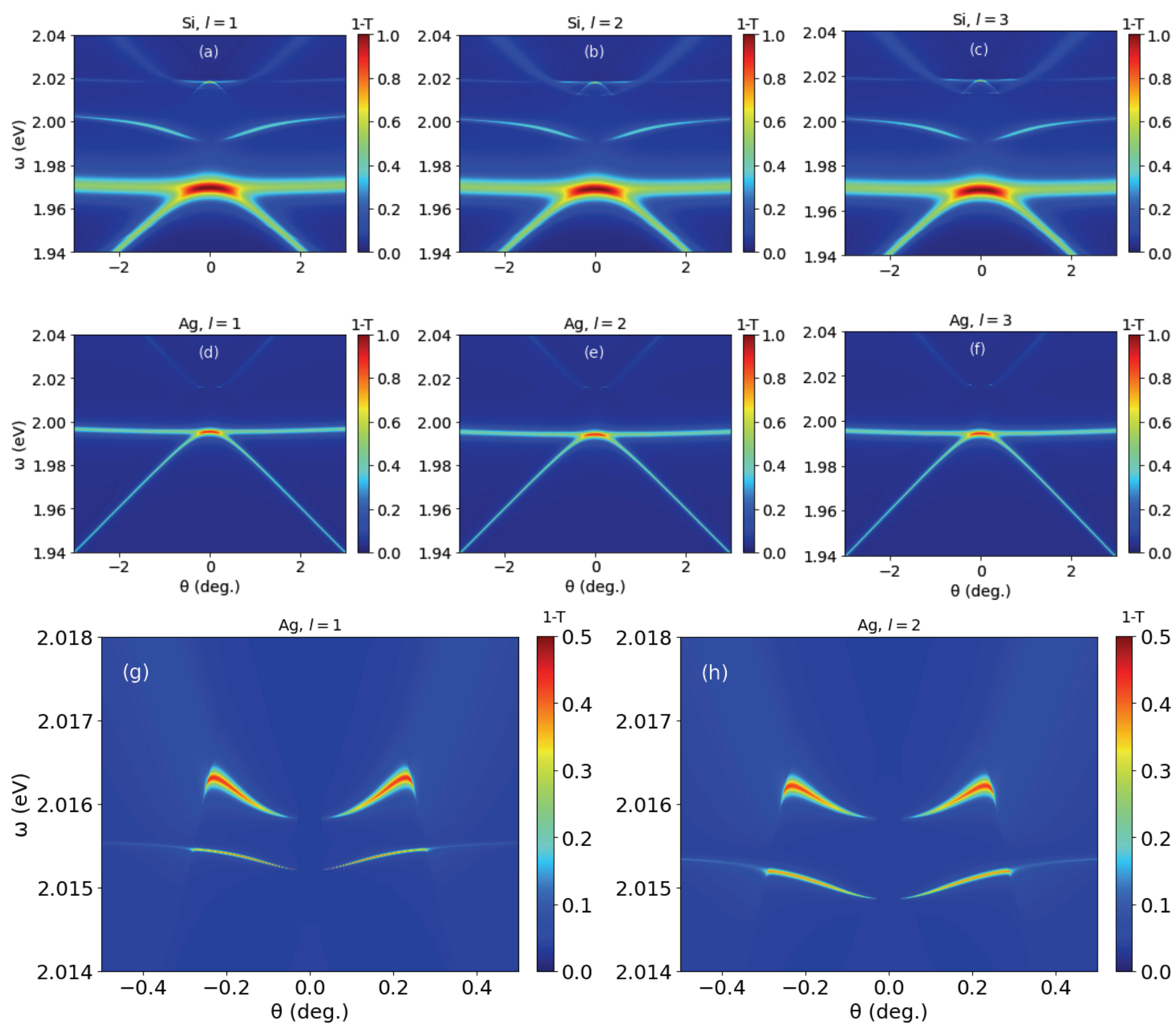

Figure S6: Color maps of the unpolarized extinction spectra as a function of the angle of incidence (in the k_x plane, $k_y = 0$) for the (a-c) Si and (d-f) Ag arrays obtained from the SMUTHI calculations including up to (a,d) dipolar ($l = 1$), (b,e) quadrupolar ($l = 2$), and (c,f) octupolar ($l = 3$) contributions. (g,h) Same as in (d,e) for the Ag array, but zooming in the (θ, ω) region near the Γ point where the quadrupolar mode emerges and nearly overlaps with one of the dipolar bands.

To shed light on the underlying physics, we carried out numerical calculations based on the free software SMUTHI (scattering by multiple particles in thin-film systems). SMUTHI is based on the T-matrix method to account for the single particle scattering and on the scattering-matrix method for the propagation through a layered medium. The scattering properties of nanodisks are actually represented by a specific T-matrix expansion for cylindrical particles, truncated at some multipolar degree l, especially accurate in determining multipolar contributions. Even if only dipolar terms are included, the interaction among particles in the array are rigorously accounted for through the scattering matrix. As can be seen in Fig. S6, all the features of the experimental extinction bands are reproduced, with a slight frequency shift and a quantitative mismatch for higher frequency modes, due to the fact that SMUTHI does not take into account absorptive losses in the dye layer. The dipolar/quadrupolar nature of the modes is confirmed in SMUTHI by calculating extinction bands up to the dipolar ($l = 1$, Fig. S6(a,d)), quadrupolar ($l = 2$, Fig. S6(b,e)), and octupolar ($l = 3$, Fig. S6(c,f)) contributions, the latter only to discard higher-order contributions. For the Ag array, we show in Fig.. S6 (g,h) the (θ, ω) region near the Γ point where the quadrupolar mode emerges and overlaps with one of the dipolar bands. Indeed, there is a significant change when including the quadrupolar contributions, especially in the lower band that shifts to lower frequencies by an amount that far exceeds its width (> 4 FWHM). As mentioned above, the

condensing mode in the case of the Si metasurface is the EQ BIC, spectrally separated from the MD modes. In the case of the Ag metasurface, the calculations confirm the spectral overlap of the EQ and MD (m_z) mode. Remarkably, SMUTHI allows to identify two related BICs separated by $\sim 10^{-3}$ eV, due to the fact that the multipoles of cylindrical shapes are very accurately accounted for. In practice, both modes ought to be mixed due to the obvious imperfections of the fabricated nanodisks. This will be further discussed below in connection with the condensation vortex shape and polarization.

S6 Near-fields and Q-factors of the BICs

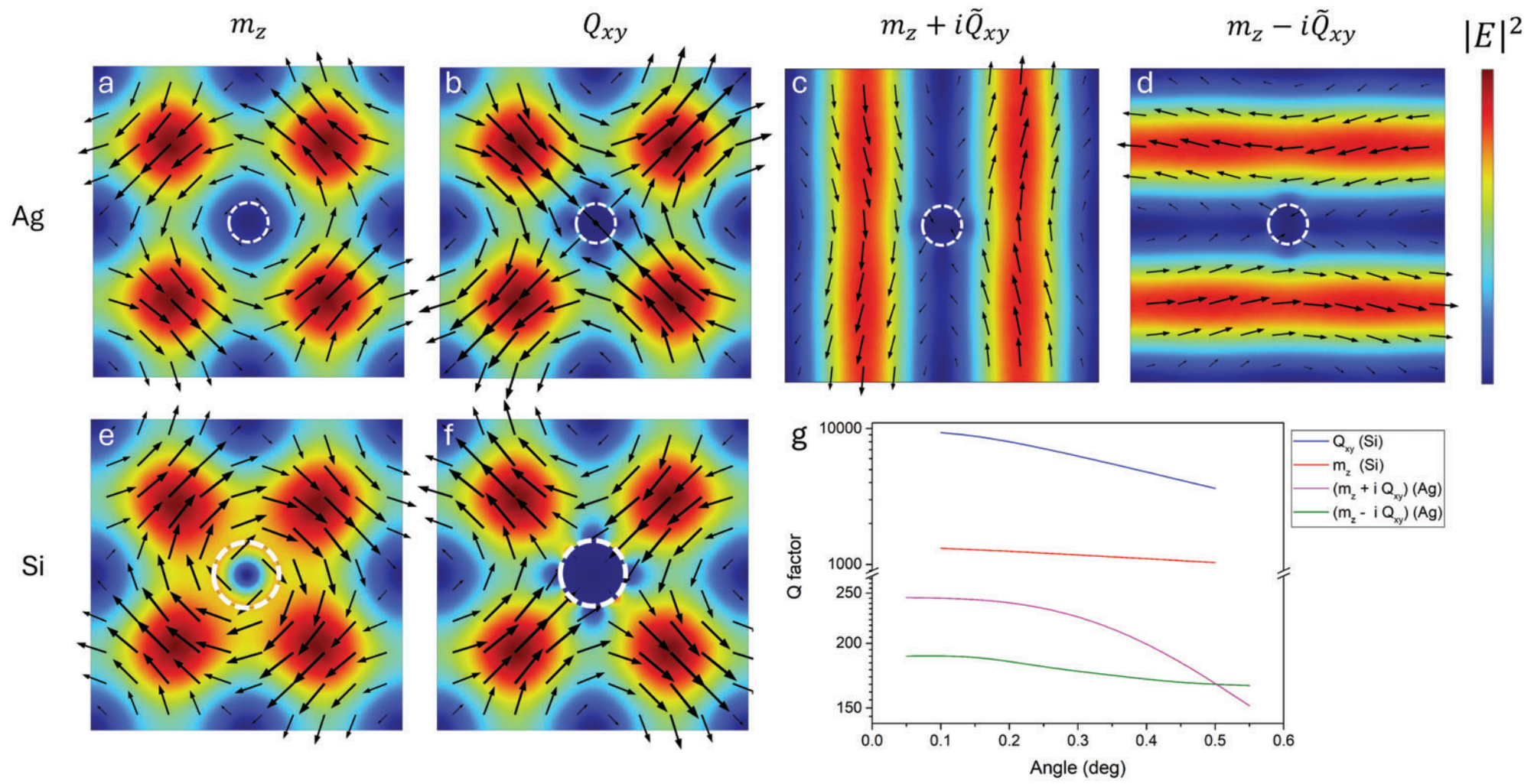

Figure S7: (a-f) COMSOL simulations of the near field distribution of the different eigenmodes for Ag (a-d) and Si (e-f) at the xy-plane crossing the nanodisk center (i.e. 45 nm from the substrate). The color scale shows the normalized value of the electric field intensity; black arrows represent the normalized in-plane components of the electric field vector (E_x, E_y). (g) Q factors of the different modes as a function of the incident angle obtained from the scattering simulations. Note that the COMSOL simulations take into account the complex values of the Si and Ag permitivities, and this prevents the Q factor from diverging at the Γ-point.

The m_z and Q_{xy} modes present a similar near-field distribution on Ag (Figures S7a and b) and Si (Figures S7e and f), with a higher field intensity within the dye layer in all cases. The main difference appears in the m_z mode on Si, which penetrates inside the Si nanodisk. This could explain why both modes are almost degenerate in the Ag metasurface, while the degeneracy is broken for Si. Figures S7c and d show the near-field patterns of the hybrid modes ($m_z + i\tilde{Q}_{xy}$) and ($m_z - i\tilde{Q}_{xy}$). The near-field intensities and polarizations resemble those of the observed far-field emission for the Ag array. Figure S7g shows the Q factors of the different modes as a function of the incident angle. In Si, the Q factor of the Q_{xy} mode is much higher, favoring the condensation in this mode. For the Ag metasurface, the ($m_z + i\tilde{Q}_{xy}$) mode (non-dispersive) presents a higher Q factor than ($m_z - i\tilde{Q}_{xy}$), thus favouring the condensation on that mode.

S7 Flat band lasing at $k_y > 0$

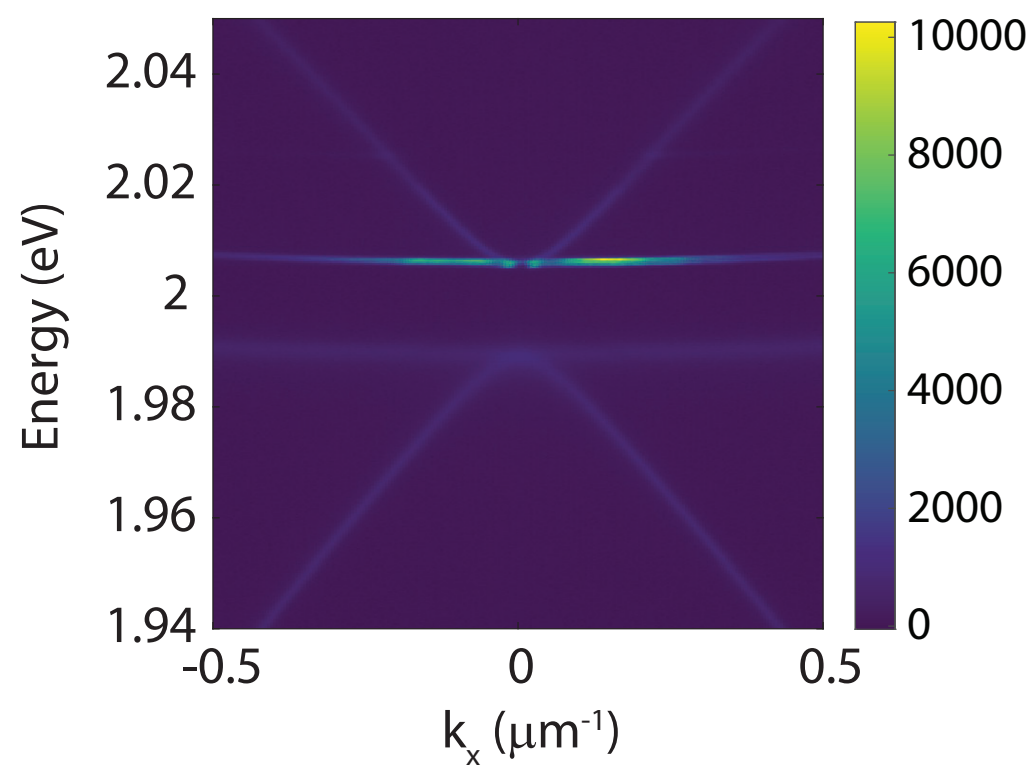

Figure S8: In the dispersion images of the condensation into the flat band of the Ag arrays, the emission from the flat bands was relatively weak, compared to the emission from the dispersive band (Fig. 3 of manuscript). The reason is that those dispersion images were taken at $k_y=0$, while the flat band emission maxima takes place at larger wave vectors following the double cross pattern (this was already clear in Fig. 4 of the manuscript). Here we image the dispersion at $k_y \sim 0.04$ rad μm^{-1} and show that there is indeed strong lasing emission from the flat band.

S8 Relation between propagation length and mode linewidth in Reciprocal space

We can show that for a condensate that decays exponentially in space with a propagation length L_p, have a linewidth $1/L_p$ in momentum space. This arises from the fact that the emission in real space and reciprocal space (in the Fourier plane) are related by the Fourier transform. If we consider a field that propagates in one direction with a propagation length L_p. The field of such exponentially decaying condensate, for positive x, is given by

$$E(x) = E_0 e^{ik_0 x} e^{\frac{-x}{2L_p}}, \tag{1}$$

giving $I(x) = |E(x)|^2 \propto e^{\frac{-x}{L_p}}$, i.e. the intensity decays to $1/e$ at $x = L_p$. In reciprocal space the field as a function of wave vector is obtained by taking the Fourier transform, assuming that E(x> 0)=0:

$$\tilde{E}(k) = \frac{E_0}{\frac{1}{2L_p} + i(k - k_0)} \tag{2}$$

The solution in intensity $I(x) = |E(x)|^2$ is

$$\tilde{I}(k) = \frac{E_0^2}{\frac{1}{2L_p}^2 + (k - k_0)^2}, \tag{3}$$

which is a Lorentzian function with a FWHM of $1/L_p$. Polaritons propagating in one direction with a decay length of L_p, will appear in reciprocal space as a peak centered at k_0 with a FWHM inversely proportional to the propagation length.

For a condensate propagating along positive and negative directions, the fields are given by:

$$\tilde{E}(k) = \int_{-\infty}^{\infty} E_0 e^{ik_0|x|} e^{\frac{-|x|}{2L_p}} e^{-ikx} dx = E_0 \int_{-\infty}^{0} e^{-ik_0 x} e^{\frac{x}{2L_p}} e^{-ikx} dx + \int_{0}^{\infty} e^{ik_0 x} e^{\frac{-x}{2L_p}} e^{-ikx} dx \tag{4}$$

Resulting in a field with two peaks at k_0 and $-k_0$:

$$\tilde{E}(k) = E_0 \left[\frac{1}{\frac{1}{2L_p} + i(k - k_0)} + \frac{1}{\frac{1}{2L_p} - i(k + k_0)} \right] \tag{5}$$

These correspond to two Lorentzian shapes in intensity as long as the peaks do not overlap. This can be shown by from the corresponding intensities by taking the absolute values squared:

$$\tilde{I}(k) = 4E_0^2 \frac{4(k_0^2 + \frac{1}{4L_p^2})}{(k^2 - k_0^2 + \frac{1}{4L_p^2})^2 + \frac{k_0^2}{L_p^2}} \tag{6}$$

For sufficiently large values of k_0, these are two well separated peaks each again with a width of $1/L_p$. This is shown if we choose $\delta k = k - k_0$, then:

$$k^2 - k_0^2 = (k_0 + \delta k)^2 - k_0^2 = 2k_0 \delta k + \delta k^2. \tag{7}$$

If we only consider the region around the peak at k_0 where $|q| >> k_0$ and k_0 is sufficiently large such that and $k_0 >> 1/L_p$ then

$$k^2 - k_0^2 = 2k_0 \delta k + \delta k^2 \simeq 2k_0 \delta k \tag{8}$$

and therefore the denominator of Eq. 10 becomes

$$(k^2 - k_0^2 + \frac{1}{4L_p^2})^2 + \frac{k_0^2}{L_p^2} \simeq (2k_0 \delta k + \frac{1}{4L_p^2})^2 + \frac{k_0^2}{L_p^2} \simeq (2k_0 \delta k)^2 + \frac{k_0^2}{L_p^2} = 4k_0^2 (\delta k^2 + \frac{1}{2L_p}^2) \tag{9}$$

This means that

$$\tilde{I}(k) \propto \frac{1}{\delta k^2 + \frac{1}{L_p^2}}, \tag{10}$$

which is again a Lorentzian lineshpae with a FWHM of $1/L_p$.

S9 Group velocities in full reciprocal space

The analysis of the group velocities as a function of only one wave vector, as was plotted in the main text Fig. 3 a and b, does not show the complete picture and can be misleading when analyzing the group velocities of the modes. While a flat band may appear along one direction, the slope of the dispersion can be high along the orthogonal direction. Resulting in a confinement along one axis only. To analyze the group velocity in both the x and y directions, we will here analyze the group velocity as a function of the in-plane wave vector in the entire plane defined by the orthogonal vectors k_x and k_y. We therefore obtain the group velocity for each combination of coordinates (k_x,k_y). We already obtained the full optical band structure of the 2D lattices in our measurements presented in Fig. S5. The first step to calculate the group velocities is to fit all the peaks in the band structure (shown in Fig. S5). We fit the 4 bands with the largest intensity in the PL emission plots. To avoid an amplification of the noise when taking the derivatives, the peak positions (k_x,k_y,λ) are first fitted with a 2D polynomial or hyperbola, depending on the shape of the dispersion. The band energies are plotted in Fig. S9 a for the Silicon array and in Fig. S9 b for the Ag array. The degeneracy of the two upper modes in the Ag array is very clear, while for the Si array, the two upper modes never intersect. From the band structure, we can calculate the group velocity for each coordinate (k_x,k_y), by taking the gradient ($v_x = \hbar \frac{dE}{dk_x}$ and $v_y = \hbar \frac{dE}{dk_y}$). It should be noted that since the BICs themselves do not emit, we do not have a data point at k_x=k_y=0 for the BIC modes. We plot the absolute normalized group velocity ($|v|$/c) = $\sqrt{v_x^2 + v_y^2}/c$ as a colormap over the dispersion of the bands in Figs. S9 c and d. So in Figs. c and d the energy of the band is still represented by the position on the z-axis, while the velocity is expressed as the color. To better understand what we are seeing, we further investigate the upper two bands that are the BICs and are most related to the condensation dynamics. In Figs. S9 e-h we plot the group velocity in the x-direction for the Si electric quadrupolar mode, the Si magnetic dipolar mode and the $(m_z - i\tilde{Q}_{xy})$ and $(m_z + i\tilde{Q}_{xy})$ modes in the silver array. It is immediately rampant that for the $(m_z - i\tilde{Q}_{xy})$ the group velocity in the x-direction is close to 0 for all plotted wave vectors, while for the other three modes, Figs e,f and g, the group velocity is zero for some specific wave vectors but large for most other wave vectors. In figs i-l we plot the group velocity in the y-direction for the same modes. Now we see that for the $(m_z - i\tilde{Q}_{xy})$ the group velocity in the y direction is not small. Therefore, while this $(m_z - i\tilde{Q}_{xy})$ mode is highly confined in the x direction, it is not in the y direction. For the $(m_z + i\tilde{Q}_{xy})$ mode, Figs. g and k, we see the opposite behavior, the group velocity is close to zero in the y-direction for all wave vectors, but large for many wave vectors in the x-direction. A fully spatially confined condensate in both the x and y directions would require low group velocities in both x and y directions. Finally, in Figs. m-p we plot the absolute group velocity of the four modes. While the group velocity of the modes is clearly different in magnitude at specific wave vectors, the difference in symmetry is very clear. The Si modes present a C_4 symmetry, while the Ag arrays have a C_2 symmetry.

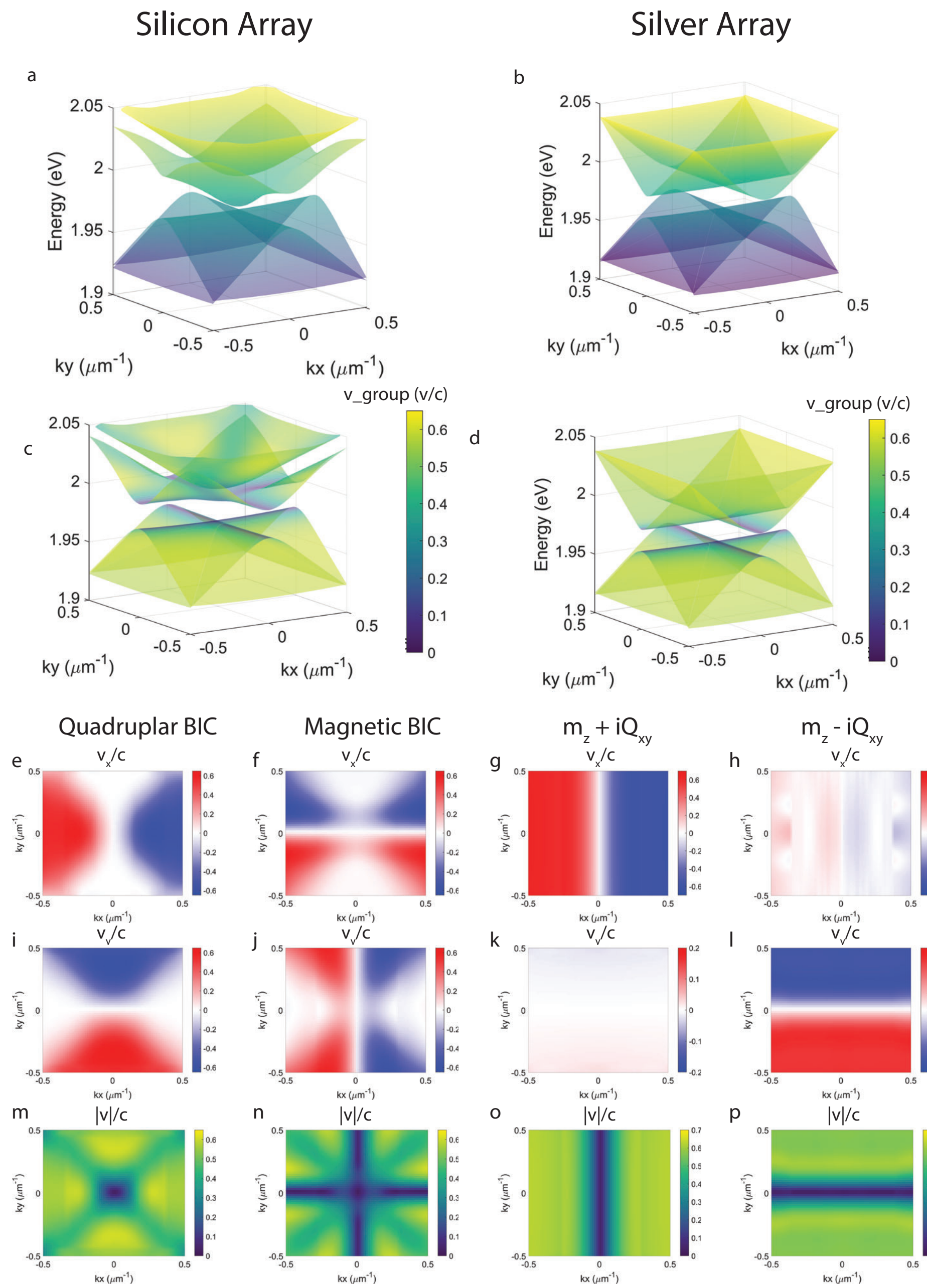

Figure S9: The photonic band structure of the Si array and Ag array, obtained from the peak maxima shown in Fig. S5. (a) and (b) show only the band structure, while in c and d, the color of the bands indicates the absolute group velocity $|v_g|$. The group velocities in the x-direction and y-direction as a function of the wave vectors are plotted in panels (e-l), for the two BIC modes in the Si and Ag arrays. (m-p) show the absolute group velocities of the BIC modes as a function of k$_x$ and k$_y$ projected on the plane.

S10 Calculated dispersions of different multipole components in the Ag array

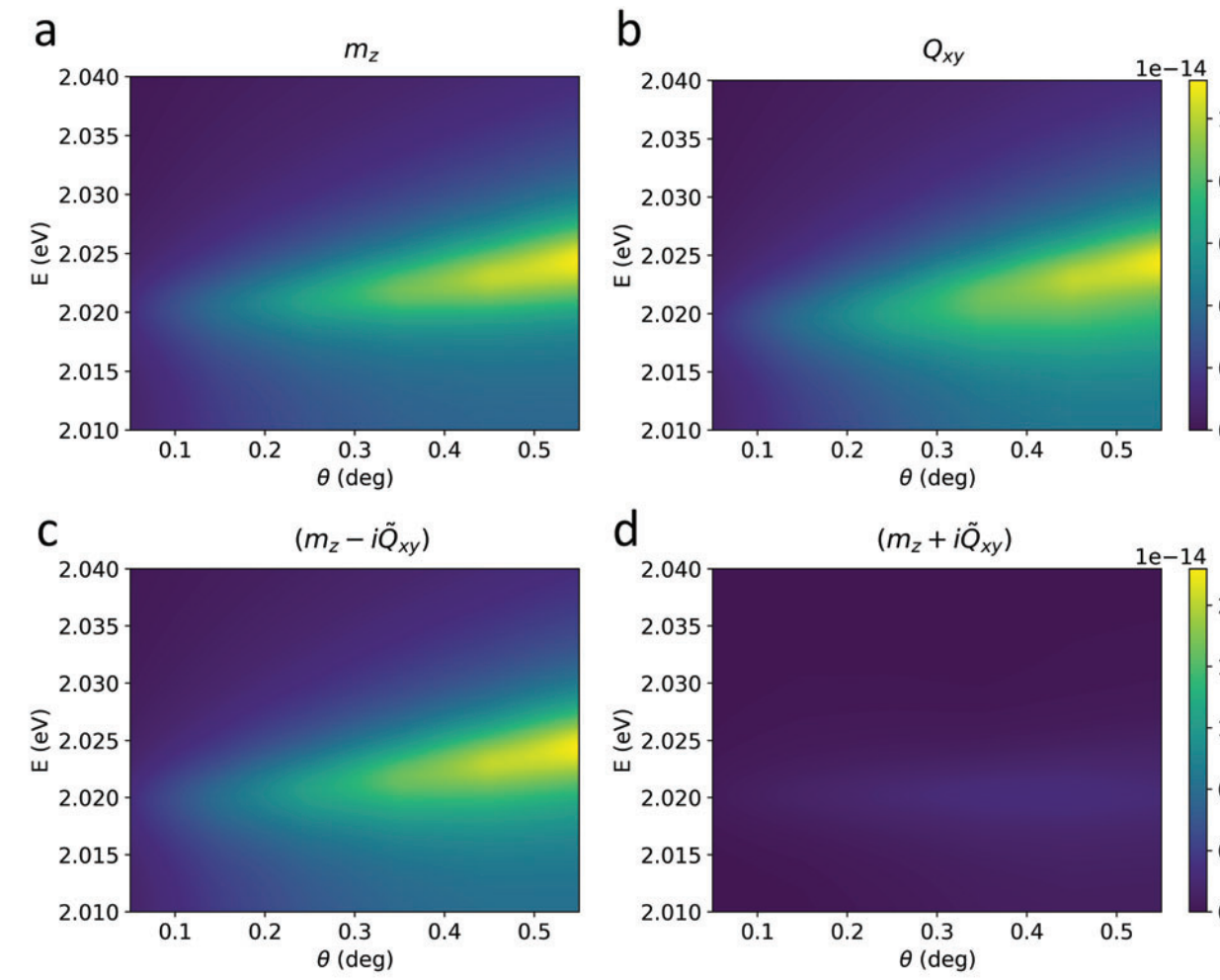

Figure S10: Calculated dispersions for different multipolar contributions in the Ag array obtained from the scattering simulations. (a) shows the m_z component and (b) the Q_{xy} component, both showing a clear angular dispersion, therefore not accounting for the observed flat-band in the Ag array. When the hybridized modes are analyzed, the dispersion of the $(m_z - i\tilde{Q}_{xy})$ (c) and $(m_z + i\tilde{Q}_{xy})$ (d) modes are plotted. The two modes are now independent, and their dispersions perfectly match the experimental observations. The $(m_z - i\tilde{Q}_{xy})$ follows the dispersive mode, while the $(m_z + i\tilde{Q}_{xy})$ accounts for the flatband.

S11 Condensation at different energies in Ag array

The condensation energy in the Si and Ag arrays with a period of 420 nm occurred at slightly lower energy for the Ag array, compared to the Si array. One may wonder if this energy difference is the reason for a different condensation mode. To exclude this possibility, we also observed the condensation in a Ag array with a smaller pitch of 415 nm such that the condensation energy in this Ag array is now slightly higher than that in the Si array (see Fig. S11).

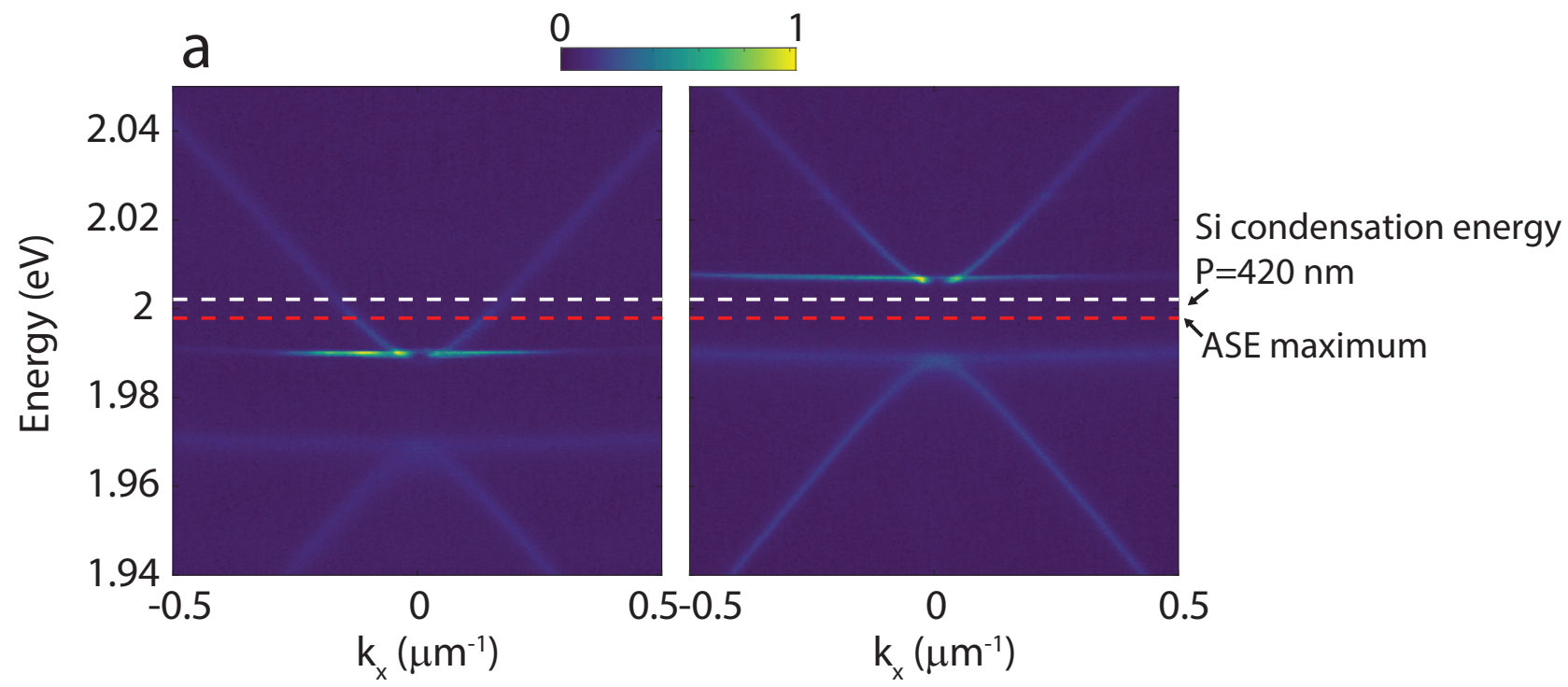

Figure S11: The condensate has been observed in arrays with different pitches. Small changes in the pitch do not affect the condensation mode. We show the formation of the condensate in an array with a 415 nm pitch (a) and an array with a pitch of 420 nm (b). The 415 nm Ag lattice has a condensation energy slightly larger than the 420 nm Si condensate (indicated with the red dashed line) and the 420 nm Ag array has an energy slightly lower than the 420 nm Si condensate. These measurements show that the different condensation modes in the Ag and Si arrays are not caused by the different energy of the modes.

S12 Real space and reciprocal space images for different orientations of the detection polarizer

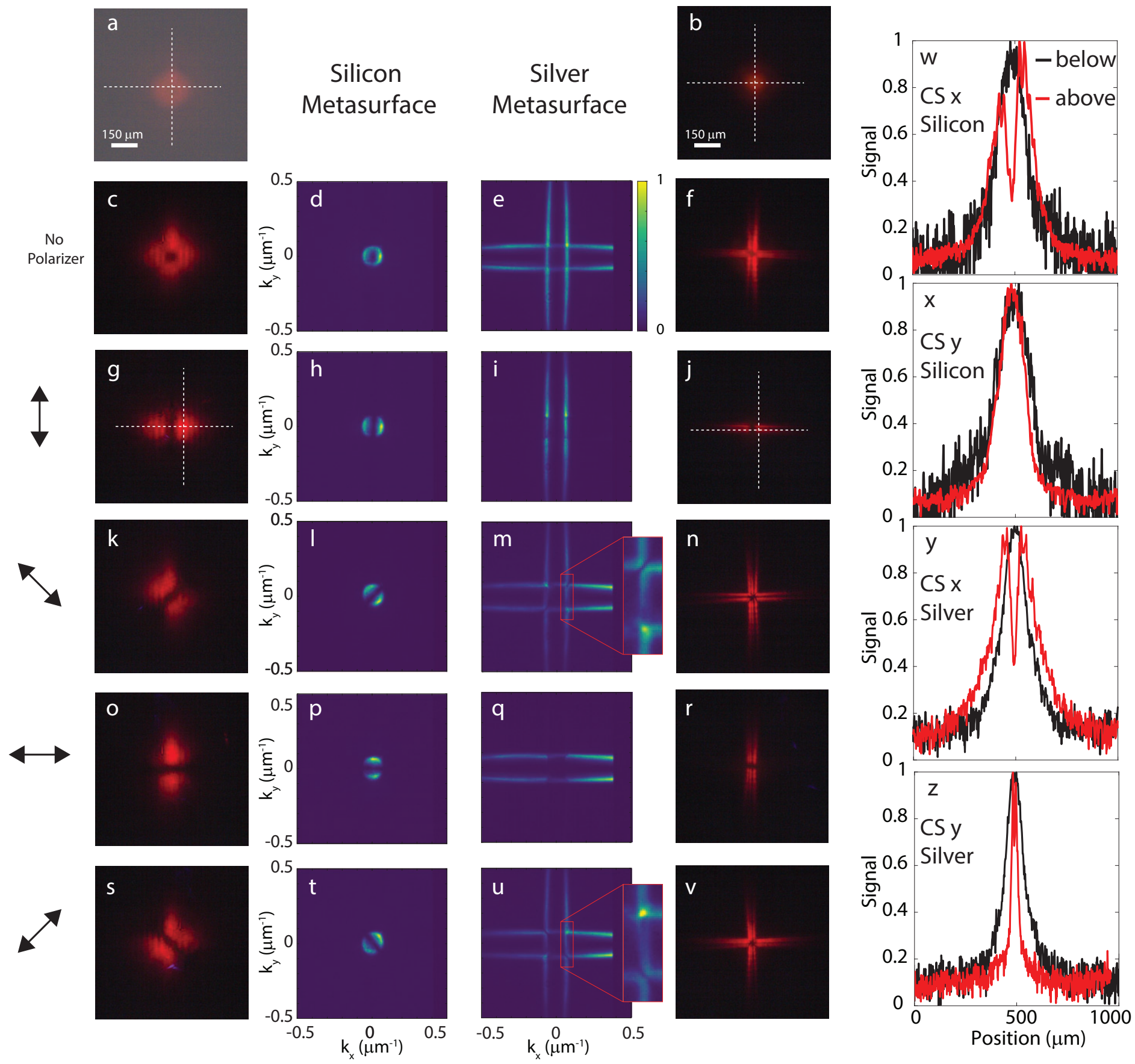

Figure S12: To investigate the condensation mode of the Ag and Si arrays, we map the condensate in both real space (red plots) and reciprocal space (green/blue plots) using different orientations of the polarizer in the detection. (a) and (b) show the area of emission below threshold. (c-f) show the emission from the condensate without a polarizer. (g-v) show the emission for different orientations of the polarizer, with the orientation indicated on the left side of the figure. The cross sections of the real-space dimensions of the condensate (along the white dashed lines) are plotted in (w-z).

S13 Hopfield coefficients

For obtaining the coupling between the cavity and the excitons from experimental data we apply a coupled oscillator model. For this model, we only analyze the dispersion of the mode turning into the Q_{xy} BIC at normal incidence. The first step is to obtain the energies of the bare cavity mode. This dispersion is obtained from the coupling of the localized Mie/plasmon resonances of the silicon/Ag particles with the in-plane diffraction orders, known as Rayleigh anomalies (RAs). The dispersion of the RAs is obtained from the geometrical parameters of the lattice:

$$E_{RA}(k_{||in})^2 = \left(\frac{\hbar c}{n_{eff}}\right)^2 \left[{k_{||in}}^2 + \left(\frac{2\pi}{a_x}p\right)^2 + \left(\frac{2\pi}{a_y}q\right)^2 + 2k_x\frac{2\pi}{a_x}p + 2k_y\frac{2\pi}{a_y}q\right], \tag{11}$$

with n_{eff} the effective refractive index of the medium, k_x and k_y the momentum of the incident wave along the x and y directions and ${k_{||in}}^2 = k_x^2 + k_y^2$ at the wavelength of the scattered waves. p and m are the diffraction orders along the x- and y-directions. For this calculation, we obtain the dispersion along k_x with $k_y = 0$ for the (p,m)=(1,0) RA. The resulting SLRs can be calculated by diagonalizing the following Hamiltonian:

$$H = \begin{bmatrix} E_{RA_1} - i\gamma_{RA_1} & 0 & g_{\mathrm{RA-LSPR}} \\ 0 & E_{RA_2} - i\gamma_{RA_2} & g_{\mathrm{RA-LSPR}} \\ g_{\mathrm{RA-LSPR}} & g_{\mathrm{RA-LSPR}} & E_L - i\gamma_L \end{bmatrix}, \tag{12}$$

where E_{RA} and γ_{RA} are the energy and losses of the RA, E_{LSPR} and γ_{LSPR} the energy and losses of the LSPR, and $g_{\mathrm{RA-LSPR}}$ the rate of interaction. The resulting eigenvalues are the SLRs. The two SLRs with the energies closest to the exciton energies are plotted as a function of the incoming wave vector with the white curves in Fig. S13. We apply another coupled oscillator model where these SLRs are coupled to the exciton resonances described by the following Hamiltonian.

$$H = \begin{bmatrix} E_{SLR_1} - i\gamma_{SLR_1} & g_{\text{SLR-SLR}} & g_{\text{X1-SLR}} & g_{\text{X2-SLR}} \\ g_{\text{SLR-SLR}} & E_{SLR_2} - i\gamma_{SLR_2} & g_{\text{X1-SLR}} & g_{\text{X2-SLR}} \\ g_{\text{X1-SLR}} & g_{\text{X1-SLR}} & E_{x1} - i\gamma_{x1} & 0 \\ g_{\text{X2-SLR}} & g_{\text{X2-SLR}} & 0 & E_{x2} - i\gamma_{x2} \end{bmatrix}, \tag{13}$$

Where E_{x1} and γ_{x1} and E_{x2} and γ_{x2} are the energies of the exciton resonance and its first vibronic replica, $g_{\text{SLR-SLR}}$ is the coupling between the 2 SLRs, $g_{\text{X1-SLR}}$ is the coupling between the SLRs and the first exciton and $g_{\text{X2-SLR}}$ the coupling between the SLR and the second exciton. The resulting eigenvalues of the 4x4 matrix are the polaritons that are plotted with the red-dashed curve in Figs. S13 a and c (and Figs. 1 e and f in the main text), the eigenvectors contain the fractions of the constituting modes. The absolute value squared of these eigenvectors are called the Hopfield coefficients. In Figs. S13 a and c, we plot the resulting eigenvalues (polaritons) with the red-dashed curves. The white-dashed curve in the same Figures indicates the polariton supporting the BIC where the condensate forms. The Hopfield coefficients of this mode are plotted as a function of the in-plane wave vector for the Si and Ag arrays in Figs. S13 b and d, respectively. The orange and blue curves represent the SLR fractions of the mode. While the exciton fractions are plotted with the yellow (main exciton transition at 2.24 eV) and purple (vibronic replica at 2.4 eV) curves. In Fig. S13 e, the polariton dispersion resulting from the coupling with the $(m_z + i\tilde{Q}_{xy})$ SLR are plotted with the red-dashed and white-dashed curves. In this case, only one SLR mode is used in the Hamiltonian of Eq. 13 (note that the white-dashed curve is fitting the faint $(m_z + i\tilde{Q}_{xy})$ and not the parabolic bright p_y dispersion. The exciton and SLR fractions of the quasi-flatband that condenses (white-dashed curve in Fig. S13 e) are shown in Fig. S13 f. The exciton fraction for both samples at the Gamma point (BIC) is approximately 10%. The parameters used to fit the dispersion are given in Table S1

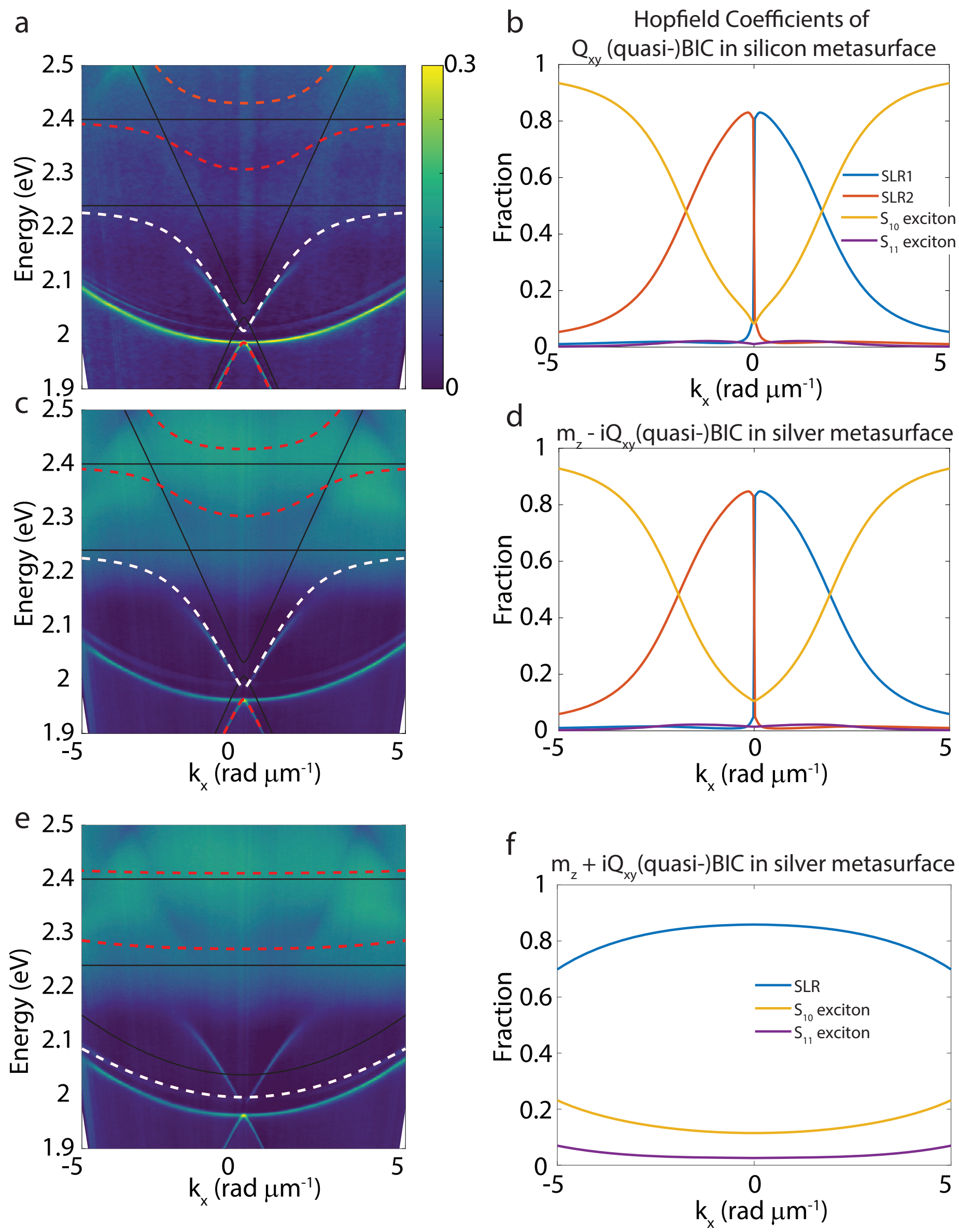

Figure S13: (a) dispersion of the polaritons in the Si array as plotted in the main text. The white-dashed curve indicates the calculated dispersion of the Q_{xy} mode where the polaritons condense at the band minimum. The Hopfield coefficients that constitute this mode are plotted in (b). (c) Dispersion of the Ag array, where the white-dashed curve indicates the $(m_z - i\tilde{Q}_{xy})$ condensation mode with in (d) the fractions of the exciton and SLRs. (e) is the same dispersion as in (c) but the red- and white-dashed curves show the polariton dispersion obtained from coupling of the $(m_z + i\tilde{Q}_{xy})$ SLR with the excitons. The exciton fractions of the polaritonic mode, plotted with the white-dashed curve, are shown in (f).

Parameter	Silicon	Silver
Coupling RA with Mie-resonacnce/LSPR		
$g_{\mathrm{RA-LSPR}}$	0.10	0.10
E_{LSPR}	2.80	2.80
γ_{LSPR}	0.05	0.07
γ_{RA}	0.002	0.002
$C_{\text{RA-LSPR}}$	0.02	0.02
Coupling SLR with excitons		
E_{X1}	2.24	2.24
E_{X2}	2.40	2.40
γ_{X1}	0.04	0.04
γ_{X2}	0.04	0.04
$g_{\text{X1-SLR}}$	0.10	0.10
$g_{\text{X2-SLR}}$	0.06	0.06
$g_{\text{SLR-SLR}}$	0.045	0.045
Lattice and optical properties		
n_{eff}	1.435	1.454
a (nm)	420	420

Table S1: Parameters used in the coupled oscillator model for Silicon and Ag. Energies and losses and coupling rates are in eV.

S14 Multipolar Decomposition Details

The multipole decomposition and calculation of the near-fields of the modes was performed with the Electromagnetic Waves in Frequency Domain module of COMSOL Multiphysics. The simulation was done for the Si array covered with perylene dye molecules in PMMA. We calculated the polarization **P** induced in the system by external plane waves impinging at different angles with TE and TM polarizations. The multipole decomposition of the modes can be calculated by integrating **P**:

$$\mathbf{p} = \int \mathbf{P} d\mathbf{r} \,, \tag{14}$$

$$\mathbf{m} = -\frac{i\omega}{2} \int (\mathbf{r} \times \mathbf{P}) d\mathbf{r} \,, \tag{15}$$

$$\hat{\mathbf{Q}} = 3 \int (\mathbf{rP} + \mathbf{Pr}) d\mathbf{r} \,, \tag{16}$$

where ω is the angular frequency, and $\mathbf{r}$ is referred to the nanodisk center. The juxtaposed terms, such as $\mathbf{rP}$, represent the dyadic product of both vectors. Note that on this basis, the electric quadrupole tensor is totally symmetric and not traceless, and the toroid-dipole terms would not appear. Higher-order contributions have been confirmed to be negligible in the considered spectral range.
